# Heterogeneity of endothelial VE-PTP downstream polarization, Tie2 activation, junctional claudin-5, and permeability in the aorta and vena cava

**DOI:** 10.1007/s00441-023-03844-9

**Published:** 2023-11-30

**Authors:** Peter Baluk, Keisuke Shirakura, Dietmar Vestweber, Donald M. McDonald

**Affiliations:** 1 Department of Anatomy, Cardiovascular Research Institute, and UCSF Helen Diller Family Comprehensive Cancer Center, University of California, 513 Parnassus Avenue, Room S1349, San Francisco, CA 94143-0452 USA; 2 Max Planck Institute for Molecular Biomedicine, Röntgenstrasse 20, Münster, 48149 Germany

**Keywords:** Blood flow, Shear stress, Adherens junctions, Tight junctions, Endothelial barrier function, Vascular permeability, Receptor-type protein tyrosine phosphatase beta (PTPRB), Immunohistochemistry, C57BL/6 mice

## Abstract

Endothelial cells of mammalian blood vessels have multiple levels of heterogeneity along the vascular tree and among different organs. Further heterogeneity results from blood flow turbulence and variations in shear stress. In the aorta, vascular endothelial protein tyrosine phosphatase (VE-PTP), which dephosphorylates tyrosine kinase receptor Tie2 in the plasma membrane, undergoes downstream polarization and endocytosis in endothelial cells exposed to laminar flow and high shear stress. VE-PTP sequestration promotes Tie2 phosphorylation at tyrosine992 and endothelial barrier tightening. The present study characterized the heterogeneity of VE-PTP polarization, Tie2-pY992 and total Tie2, and claudin-5 in anatomically defined regions of endothelial cells in the mouse descending thoracic aorta, where laminar flow is variable and IgG extravasation is patchy. We discovered that VE-PTP and Tie2-pY992 had mosaic patterns, unlike the uniform distribution of total Tie2. Claudin-5 at tight junctions also had a mosaic pattern, whereas VE-cadherin at adherens junctions bordered all endothelial cells. Importantly, the amounts of Tie2-pY992 and claudin-5 in aortic endothelial cells correlated with downstream polarization of VE-PTP. VE-PTP and Tie2-pY992 also had mosaic patterns in the vena cava, but claudin-5 was nearly absent and extravasated IgG was ubiquitous. Correlation of Tie2-pY992 and claudin-5 with VE-PTP polarization supports their collective interaction in the regulation of endothelial barrier function in the aorta, yet differences between the aorta and vena cava indicate additional flow-related determinants of permeability. Together, the results highlight new levels of endothelial cell functional mosaicism in the aorta and vena cava, where blood flow dynamics are well known to be heterogeneous.

## Introduction

Multiple levels of heterogeneity of endothelial cells are evident in mammalian blood vessels. One level consists of segment-specific properties of endothelial cells in arteries, veins, and intervening parts of the microvasculature - arterioles, capillaries, postcapillary venules, and collecting venules (Aird 2012; Johnson 2008; Majno 1965; Simionescu and Simionescu 1984). Among other differences, the shape of endothelial cells has long been recognized to vary along these vascular segments (Casparis 1918; Dela Paz and D'Amore 2009; McDonald 1994). Another level of heterogeneity is found in organ-specific differences in endothelial cells that reflect adaptations to the specialized functions of each organ (Augustin and Koh 2017; Minami et al. 2019; Richards et al. 2021). Additional endothelial cell heterogeneity is found in differences revealed by single-cell analysis of gene expression (scRNA-seq) (Becker et al. 2022; Betsholtz 2022; He et al. 2018; Huang et al. 2021; Kalluri et al. 2019; Kalucka et al. 2020; Vanlandewijck et al. 2018).

Further endothelial cell heterogeneity results from differences in blood flow and luminal shear stress. Endothelial cells in regions of arteries with predominantly laminar blood flow and high shear stress, as in the descending thoracic aorta, differ from those in the inner curvature of the aortic arch and other regions exposed to disturbed flow and low shear stress (Davies et al. 2010; Gimbrone and Garcia-Cardena 2013). Consequences of turbulent or oscillatory flow include endothelial leakiness and susceptibility to development of atherosclerosis (Davies et al. 2010; Gimbrone and Garcia-Cardena 2013). The heterogeneity of blood flow and wall shear stress in the aorta is documented by in vivo measurements supported by computational models (Huo et al. 2008; Mohamied et al. 2017; Peiffer et al. 2013). Although the cardiac connection, arch, and branch points of the aorta have the greatest flow disturbance, all regions are exposed to pulsations and changing flow patterns (Huo et al. 2008; Mohamied et al. 2017; Peiffer et al. 2013). With this perspective, the functional properties and anatomical distribution of endothelial cell subsets identified by sc-RNA-seq are now beginning to be mapped in the normal mouse aorta (Engelbrecht et al. 2020; Kalluri et al. 2019).

In previous work, we found that vascular endothelial protein tyrosine phosphatase (VE-PTP), a receptor type tyrosine phosphatase, has a heterogeneous distribution in endothelial cells of the aorta (Shirakura et al. 2023). The distribution of VE-PTP influences the phosphorylation of Tie2 receptors in the plasma membrane and thereby endothelial barrier stability (Frye et al. 2015; Winderlich et al. 2009). In regions of the aorta exposed to high average shear stress, VE-PTP is polarized toward the downstream pole of endothelial cells, where it is sequestered from the plasma membrane by endocytosis (Shirakura et al. 2023). Caveolin-1, phosphorylated endothelial nitric oxide synthase (eNOS-pS1177), and transient receptor potential vanilloid-type 4 (Trpv4) ion channels have also been reported to undergo flow-dependent downstream polarization in aortic endothelial cells (Hong et al. 2023). Downstream polarization of VE-PTP in aortic endothelial cells reflects the flow-dependent redistribution of VE-PTP identified in cultured cells (Mantilidewi et al. 2014). Sequestration of VE-PTP away from Tie2 leads to greater Tie2 phosphorylation at tyrosine 992 (Tie2-pY992) and endothelial barrier tightening (Shirakura et al. 2023). In contrast, dispersal of VE-PTP in the plasma membrane favors Tie2 dephosphorylation, increased macromolecular permeability, and atherogenesis in regions exposed to disturbed flow (Shirakura et al. 2023).

Based on the evidence of polarization and heterogeneity of VE-PTP and Tie2-pY992 in aortic endothelial cells, we then sought to determine whether similar features were present in microvascular endothelial cells. However, the small size, tight curvature, and tortuosity of the vessels prevented meaningful visualization by confocal microscopy. Therefore, in the present study, we characterized the heterogeneity of VE-PTP, Tie2-pY992, and endothelial barrier proteins in aortic endothelial cells that have well-documented regional differences, relevance to atherogenesis, and could be visualized in large areas of endothelium in 3-dimensional whole mounts. The descending thoracic aorta was selected as a region where laminar blood flow occurs but is perturbed by cardiac pulsatility, intercostal arterial branches, and other factors. The vena cava, which could also be visualized in 3-dimensional whole mounts, was used for comparison to determine whether the observed features were unique to the aorta.

Anatomically standardized regions of whole mounts of aorta and vena cava stained for VE-PTP and related proteins were examined to: (1) measure the spatial heterogeneity of VE-PTP in the plasma membrane and cytoplasm of endothelial cells; (2) compare the heterogeneity of VE-PTP to the distribution of Tie2-pY992 as an index of endothelial barrier stability; (3) determine whether the heterogeneity of VE-PTP is accompanied by corresponding variability in claudin-5 at tight junctions or vascular endothelial cadherin (VE-cadherin) at adherens junctions; and (4) relate these features to focal differences in permeability reflected by accumulations of extravasated endogenous IgG.

The study revealed that VE-PTP polarization in endothelial cells had a mosaic pattern in the thoracic aorta and vena cava, where groups of endothelial cells with polarized VE-PTP were mixed with others having little or none. In the aorta, Tie2-pY992 and claudin-5 had similar mosaic patterns, but total Tie2 was widespread, and VE-cadherin bordered all endothelial cells. The mosaic patterns of Tie2-pY992 and claudin-5 correlated with VE-PTP polarization but not with patchy accumulations of extravasated IgG, likely due to slow clearance of IgG from the aortic wall. VE-PTP and Tie2-pY992 also had mosaic patterns in the vena cava, but claudin-5 was nearly absent and extravasated IgG was ubiquitous. These endothelial cell heterogeneities accompany well documented blood flow and shear stress dynamics in the aorta and vena cava.

## Materials and methods

### Mice

Approximately equal numbers of male and female wild-type C57BL/6 mice, bred at UCSF or Max Planck Institute for Molecular Biomedicine or purchased from The Jackson Laboratory, were anesthetized with ketamine (87 mg/kg) and xylazine (10 mg/kg) injected intraperitoneally at age 8 to 10 wk. Tissues were fixed by perfusion of 1% paraformaldehyde (PFA) through the heart; then the aorta and vena cava were removed as whole mounts and further fixed for 1 h in 1% PFA for immunohistochemical staining. All studies were approved by the Institutional Animal Care and Use Committee of the University of California, San Francisco, USA (Approval AN183863-02) or the Landesamt für Natur, Umwelt und Verbraucherschutz Nordrhein-Westfalen, Germany (Approval 81-02.04.2020.A023).

### Immunohistochemical staining

After fixation, the aorta and vena cava were washed with phosphate buffered saline (PBS), incised longitudinally to create flattenable whole mounts, and permeabilized in PBS with 0.3% TritonX-100 for 1 h. The specimens were then incubated in primary antibodies diluted in 10% donkey serum in PBS with 0.3% TritonX-100 at room temperature overnight, washed the next day, and stained overnight in secondary antibodies in PBS (Shirakura et al. 2023). TritonX-100 was omitted from staining of non-permeabilized aortas. After washing, the vessels were mounted in Vectashield with 50 mg/mL DAPI (Vector Laboratories) or Dako mounting medium on glass slides with the endothelium facing the cover slip.

### Antibodies

Primary antibodies used for immunohistochemical staining included: rat monoclonal anti-mouse VE-PTP (clone 109.1, 1:500, (Baumer et al. 2006)); goat polyclonal anti-mouse Tie2 (AF762, 1:500, R&D Systems); rabbit polyclonal tyrosine-992 phosphorylated anti-mouse Tie2 (Tie2-pY992, AF2720, 1:500, R&D Systems); rabbit polyclonal anti-mouse claudin-5 (34-1600, 1:1000, Invitrogen); rabbit monoclonal anti-mouse VE-cadherin (clone Vii37, MABT886, 1:1000, Sigma); rat monoclonal anti-mouse VE-cadherin (BD 550548, 1:1000, BD Biosciences); goat polyclonal anti-mouse VE-cadherin (AF1002, 1:1000, R&D Systems); rabbit polyclonal anti-human von Willebrand factor (vWF, A0082, 2 μg/ml, Dako); Cy5-conjugated donkey anti-mouse IgG (AP192C, 1:500, Sigma); and chicken polyclonal anti-human lamin A/C (NBP-2-25152, 1:500, Novus). Secondary antibodies were conjugated to Cy3, Alexa Fluor 488, Alexa Fluor 568, or Alexa Fluor 647 (Jackson ImmunoResearch).

### Confocal microscope calibration and imaging

Red-green-blue (RGB) color images (1024 × 1024 pixels) were obtained with a Zeiss LSM 510 or LSM 880 confocal microscope with objective lenses that gave the following pixel sizes and image areas: Zeiss ×100 NA 1.4 oil immersion, pixel size 0.124 µm, image area 0.0161 mm^2^; Zeiss ×63 NA 1.4 oil immersion, pixel size 0.132 µm, image area 0.0182 mm^2^; and Zeiss ×40 NA 1.0, pixel size 0.311 µm, image area 0.101 mm^2^. Images were obtained of anatomically defined regions of flattened whole mounts of thoracic aorta located 100–400 µm upstream of intercostal artery ostia and inferior (caudal) vena cava midway between the right atrium and the diaphragm. Inner and outer curvatures of the arch were also imaged in some aortas. Specimens were oriented with the direction of blood flow from left to right.

### VE-PTP in endothelial cells of aorta and vena cava

The size, number, and distribution of VE-PTP particles in aorta and vena cava whole mounts stained for VE-PTP and VE-cadherin were measured in confocal microscopic images (×100 objective) of endothelial cells by using the Analyze Particles tool of ImageJ/Fiji (https://imagej.net). VE-PTP fluorescence above an intensity threshold of 60 (range 0-255) was analyzed in binary images of the VE-PTP channel (ImageJ/Fiji > Process > Binary > Convert to Mask). VE-PTP particles in three size groups (1 ≤ 10 pixels, 11 ≤ 100 pixels, and > 100 pixels) were counted in each image (ImageJ/Fiji > VE-PTP binary image > Analyze Particles) and expressed as particles per endothelial cell or fractional area (area density, %). Diameters of the three VE-PTP size groups (≤ 0.4 µm, 0.4 ≤ 1.4 µm, and > 1.4 µm) were calculated from pixel size and number assuming circularity. VE-PTP polarization was quantified by counting particles in the three size groups in the upstream and downstream halves of 8 uniformly distributed endothelial cells in each image and was expressed as the percent in the downstream half. Endothelial cells in each image were counted in the VE-cadherin channel (binary image threshold 60–100, mean 38 cells/image), and the proportion of cells with VE-PTP particles of the three sizes was calculated. Means ± standard error of the mean (SEM) were calculated from values from 12 images of aorta and 12 images of vena cava from 6 mice unless specified otherwise.

### VE-PTP colocalized with VE-cadherin at endothelial cell junctions

VE-PTP at endothelial cell junctions in the aorta and vena cava was identified by colocalization with VE-cadherin in binary images made from ×100 confocal microscopic images (ImageJ/Fiji > Colocalization plugin). Junctional VE-PTP was calculated as percent of total VE-PTP. The distribution of VE-PTP in relation to VE-cadherin at junctions was also analyzed by plotting van Steensel's Cross-correlation Coefficients (CCF). In this procedure, 40 CCF values were calculated sequentially as the VE-PTP channel was shifted stepwise by 1 pixel increments from -20 to +20 pixels in relation to the VE-cadherin channel (ImageJ/Fiji > JACoP plugin) (Cordelières and Bolte 2008; van Steensel et al. 1996). Plots of CCF values displayed the distribution of VE-PTP in relation to VE-cadherin. Positive pixel shift values ($\delta$x) indicated that non-colocalized VE-PTP in the cytoplasm was to the left (upstream) of colocalized VE-PTP in the plasma membrane. The CCF peak marked maximal colocalization in the plasma membrane. Lower values on the left reflected decreasing colocalization as cytoplasmic VE-PTP increased.

### VE-PTP staining with or without permeabilization

The location of VE-PTP within endothelial cells was determined by comparing ×63 images of aortas stained with or without permeabilization by TritonX-100 (see Immunohistochemical staining). vWF was stained with VE-PTP as a positive control for intracellular staining. All specimens were subsequently stained for VE-cadherin in the presence of TritonX-100. The number and size of VE-PTP particles in stained whole mounts were measured above a fluorescence intensity threshold of 100 and expressed per endothelial cell and as area densities, as described in the paragraph entitled *VE-PTP in endothelial cells of aorta and vena cava*. vWF and VE-cadherin area densities were measured above a threshold of 60. Measurements were made on 12 confocal images of 6 permeabilized aortas and 12 images of 6 non-permeabilized aortas.

### Relation of Tie2-pY992 to total Tie2

Relative amounts and distributions of Tie2-pY992 and total Tie2 were compared in ×100 images of endothelial cells in the aorta and vena cava. Overall area densities and percent colocalization of Tie2-pY992 and Tie2 fluorescence above intensity thresholds of 60–100 were measured in binary images created from confocal microscopic images (ImageJ/Fiji > Colocalization plugin).

### Relation of Tie2-pY992 to VE-PTP polarization

The amount and distribution of Tie2-pY992 and VE-PTP were compared in ×100 images of endothelial cells of the thoracic aorta and vena cava stained with pairs of primary antibodies. Correlation of area densities of overall staining was assessed by linear regression analysis. Correlation of Tie2-pY992 at cell junctions with VE-PTP particles > 100 pixels (> 1.4 µm in diameter) was similarly tested. Tie2-pY992 at cell junctions, sampled by using VE-cadherin staining as a binary mask, was expressed as percent of overall Tie2-pY992 staining. Measurements were made of 12 regions of each aorta and vena cava from 5–6 mice.

### Relation of claudin-5 to VE-PTP polarization

Area densities of claudin-5 and VE-PTP were measured in binary images (threshold 60) prepared from ×63 or ×100 confocal microscopic images of thoracic aorta and vena cava by using ImageJ/Fiji. Correlation of claudin-5 to VE-PTP polarization was assessed by comparing the respective area densities in 32 regions of ×100 images of aortas of two mice by linear regression analysis. Area densities of claudin-5 and VE-PTP were also measured in sequential 30–36 250 × 1500-pixel regions of ×63 confocal image panoramas of the entire circumference of three additional aortas and compared by linear regression analysis and fluorescence intensity plot profiles.

### Extravasated IgG in aorta and vena cava

Sites of plasma extravasation in the aorta and vena were assessed by measuring the area density of accumulations of endogenous IgG (fluorescence intensity threshold 60) in ×100 images of the vessel wall. The relationship of IgG accumulations to Tie2-pY992 or claudin-5 was assessed by comparing IgG fluorescence in regions with or without Tie2-pY992 or claudin-5 staining (ImageJ/Fiji > Colocalization plugin). Amounts of IgG in the two regions were compared by linear regression analysis, where IgG area densities in regions with or without Tie2-pY992 were compared to overall IgG and to each other in 11 regions of each aorta and vena cava from 6 mice.

### Statistical analysis

Mice of both genders matched for age were randomly assigned to groups. Group size determined from pilot studies by power analysis ranged from 5–16 mice to achieve statistical power of 0.8 and *P* value of < 0.05. Values are expressed as mean ± SEM. The number of images, number of mice per group, and statistical significance of differences are shown in figure legends. *P*-values < 0.05 were considered statistically significant. Heterogeneity was assessed by calculating coefficients of variation (standard deviation divided by mean). Differences between two groups were assessed by two-tailed Student's *t*-test and among three or more groups by one-way or two-way ANOVA followed by the Tukey test of multiple comparisons (Prism, GraphPad). Correlation of paired datasets was assessed by simple linear regression (Prism). Frequency distributions of VE-PTP particle sizes in permeabilized and non-permeabilized aortas and plot profiles of VE-PTP and claudin-5 area densities around the aortic circumference were compared by the Kolmogorov-Smirnov two-sample test.

## Results

### Heterogeneity of VE-PTP polarization in endothelial cells of aorta and vena cava

VE-PTP in regions of the aorta with laminar flow and high shear stress is polarized to the downstream half of endothelial cells (Shirakura et al. 2023). VE-PTP polarization occurs in the descending thoracic aorta and outer curvature of the aortic arch but not in the inner curvature where flow is disturbed (Shirakura et al. 2023).

Even in the descending thoracic aorta, the amount of VE-PTP polarization varies from region to region (Shirakura et al. 2023). To better understand the heterogeneity, we analyzed the distribution of VE-PTP in defined regions of this part of the aorta, where cardiac pulsatility, intercostal branches, and other factors perturb laminar flow (Mohamied et al. 2017). For comparison, we made similar observations in the vena cava, where the pressure is much lower, flow is slower, and pulsations reflect both cardiac and respiratory rhythms (Joseph et al. 2020; Mesin et al. 2020; Tabima et al. 2010).

**Fig. 1:**
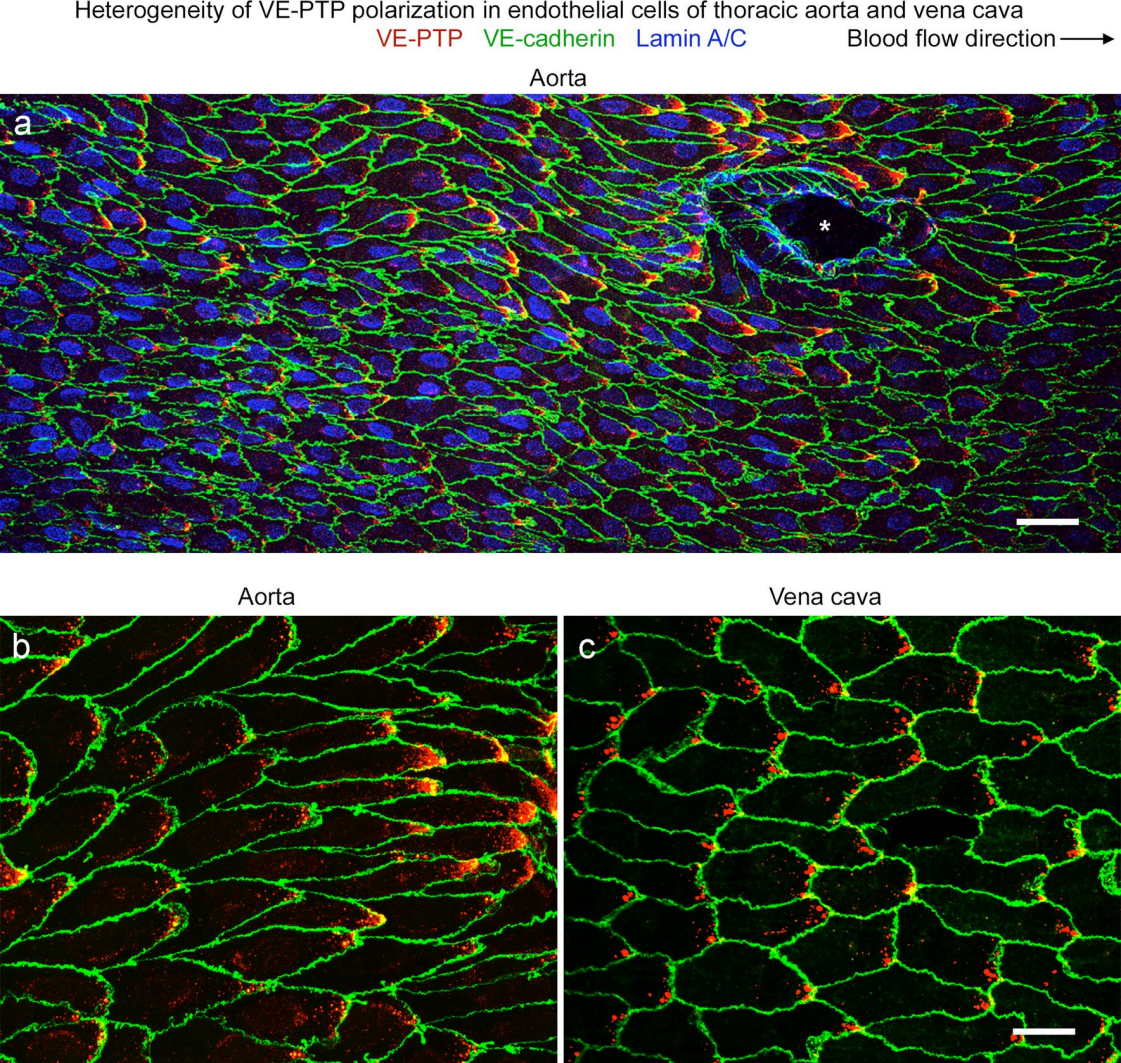
Heterogeneity of VE-PTP downstream polarization in aorta and vena cava. **a**–**c** Confocal microscopic images of immunohistochemical staining for VE-PTP (red), VE-cadherin, (green), and nuclei (blue, lamin A/C) of endothelial cells in the descending thoracic aorta near an intercostal artery ostium (asterisk) (**a**,** b**) and inferior vena cava between the right atrium and diaphragm (**c**). Blood flow is left to right. VE-PTP is concentrated in the downstream half of endothelial cells, but the amount varies from cell to cell. A conspicuous patch of VE-PTP is located near the downstream tip of some endothelial cells but not in others. Downstream concentrations of VE-PTP are larger in the aorta (**a**,** b**) but are more frequent in the vena cava (**c**). Scale bars: **a** 50 µm, **b**,** c** 20 µm

Examination of the endothelium of the descending thoracic aorta in normal mice revealed a mosaic of VE-PTP staining, where regions with VE-PTP polarization were next to regions with little or none (Fig. 1a, b). VE-PTP polarization was prominent in endothelial cells around some intercostal ostia (Fig. 1a). Otherwise, the heterogeneity of VE-PTP polarization in endothelial cells lacked apparent anatomical landmarks and resembled the variability in wall shear stress found in functional studies (Mohamied et al. 2017).

The inferior vena cava of the same mice also had regions where VE-PTP was polarized to the downstream half of endothelial cells (Fig. 1c). The polarization varied from region to region, but the pattern was more uniform and the patches of VE-PTP were smaller than in the thoracic aorta (Fig. 1b, c).

### Heterogeneity of VE-PTP particle size and number in endothelial cells

**Fig. 2:**
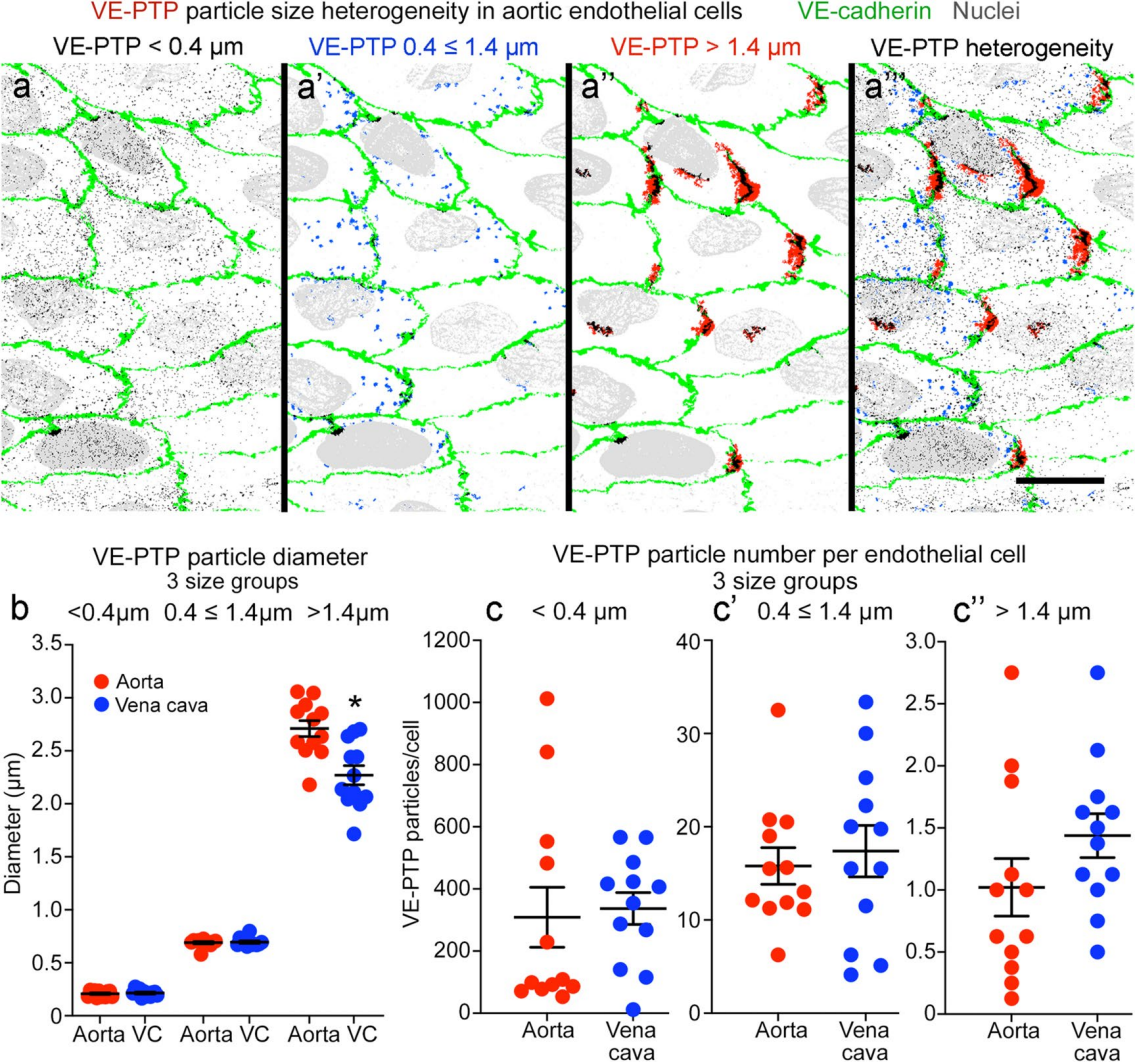
Heterogeneity of VE-PTP particle size and number in aorta and vena cava. **a**–**a’’’** Color-coded binary images made from confocal microscopic images of aortic endothelial cells to show 3 sizes of VE-PTP particles separately and together. Blood flow left to right. The smallest particles, < 0.4 µm in diameter (**a**, black, 1 < 10 pixels), are widely scattered, whereas those 0.4 ≤ 1.4 µm in diameter (**a’**, blue, 11 ≤ 100 pixels) resemble endosomes and are most abundant in the downstream half of endothelial cells. The largest VE-PTP particles, > 1.4 µm in diameter (**a’’**, red, > 100 pixels), partially overlap VE-cadherin (green) at the downstream cell tip. VE-PTP colocalized with VE-cadherin is black. Scale bar: 20 µm. **b** Size comparison of 3 groups of VE-PTP particles in endothelial cells of the aorta and vena cava (VC). VE-PTP particles in the > 1.4 µm group had the greatest variability in size and were significantly larger in the aorta than vena cava. *P < 0.05, by one-way ANOVA followed by Tukey’s multiple comparison test. Mean ± SEM. *n* = 12 images from 6 mice/group. **c**–**c’’** Comparison of number of 3 sizes of VE-PTP particles per endothelial cell. Region-to-region heterogeneity is evident in the aorta and vena cava. Mean ± SEM, *n* = 12 images from 6 mice/group

VE-PTP immunoreactivity in endothelial cells was particulate and ranged in size (Fig. 2a–a’’’). The particles were apportioned into three size groups based on the number of pixels per particle (1 ≤ 10, 11 ≤ 100, or > 100 pixels). Particles in these groups had diameters of ≤ 0.4 µm, 0.4 ≤ 1.4 µm, or > 1.4 µm. The size groups proved informative. The smallest particles were evenly dispersed (Fig. 2a), but larger particles were concentrated in the downstream half of endothelial cells (Fig. 2a’), and the largest particles were restricted to the downstream cell tip (Fig. 2a’’).

Small VE-PTP particles were similar in size in the aorta and vena cava, but those larger than 1.4 µm averaged 16% smaller in the vena cava (Fig. 2b). The homogeneity of VE-PTP particle size from region to region was reflected by small coefficients of variation, which in the aorta were 14% for the ≤ 0.4 size group, 5% for the 0.4 ≤ 1.4 µm group, and 10% for the > 1.4 µm group. Corresponding coefficients of variation for the vena cava were 14%, 6%, and 14% (Fig. 2b).

However, VE-PTP particles were much more heterogeneous in number than in size (Fig. 2c–c’’). Log differences in number were found among the three size groups, and the number of VE-PTP particles per endothelial cell varied from region-to-region of aorta and vena cava (Fig. 2c–c’’). The coefficient of variation was 118% for the ≤ 0.4 µm size group, 45% for the 0.4 ≤ 1.4 µm group, and 62% for the > 1.4 µm group in the aorta. The corresponding coefficients of variation in the vena cava were 57%, 59%, and 40%.

**Fig. 3:**
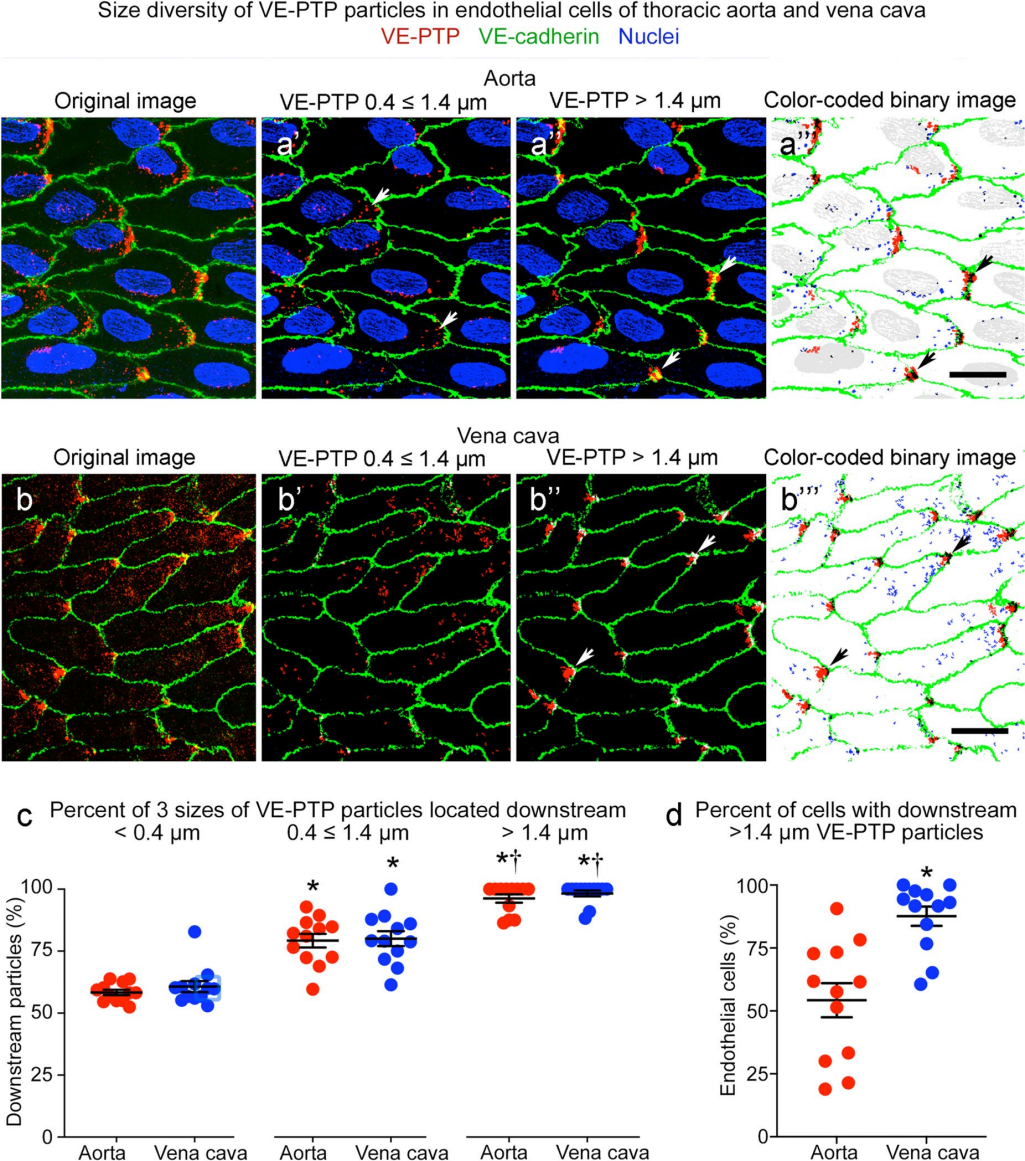
Size differences in VE-PTP particles polarized downstream in aorta and vena cava. **a**–**a’’’** Confocal microscopic images of VE-PTP (red), VE-cadherin (green), and nuclei (blue, lamin A/C) in thoracic aorta comparing the original image (**a**) to binary images of VE-PTP particles with diameters of 0.4 ≤ 1.4 µm (**a’**) or > 1.4 µm (**a’’**) and color-coded composite of the 0.4 < 1.4 µm (blue) and > 1.4 µm (red) binary images (**a’’’**). Blood flow left to right. VE-PTP particles 0.4 ≤ 1.4 µm in diameter are scattered in the downstream cytoplasm, whereas VE-PTP particles > 1.4 µm are concentrated at the downstream cell tip (arrows). **b**–**b’’’** Images of vena cava stained as in (**a**–**a’’’**). Arrows mark VE-PTP at the downstream cell tip. Scale bars: 10 µm.** c** Percentages of 3 sizes of VE-PTP particles in the downstream half of endothelial cells in the aorta and vena cava. *P < 0.05 compared to < 0.4-µm group; †*P* < 0.05 compared to 0.4 ≤ 1.4 µm group, by one-way ANOVA followed by Tukey’s multiple comparison test. Mean ± SEM, *n* = 12 images from 6 mice/group. **d** Dot plots showing percentages of endothelial cells with > 1.4 µm VE-PTP particles with significantly larger values for the vena cava than for the aorta. *P < 0.05 by Student’s *t* test. Mean ± SEM, *n* = 12 images from 6 mice/group

The smallest VE-PTP particles (≤ 0.4 µm) were nearly uniformly distributed in endothelial cells in the aorta and vena cava (Figs. 2a and 3a, b), but larger particles were significantly more numerous downstream than upstream (Figs. 2a’, a’’ and 3a’–a’’’, b’–b’’’). About 60% of particles ≤ 0.4 µm in diameter, 80% of particles 0.4 ≤ 1.4 µm in diameter, and 97% of those > 1.4 µm were in the downstream half of endothelial cells in both vessels (Fig. 3c). Almost all VE-PTP particles > 1.4 µm in diameter were at the downstream cell tip (Fig. 3a’’, a’’’, b’’, b’’’). On average, these large particles were present in 54% of endothelial cells in the aorta and 88% of endothelial cells in the vena cava (Fig. 3d).

### Heterogeneity of VE-PTP downstream polarization in endothelial cells

**Fig. 4:**
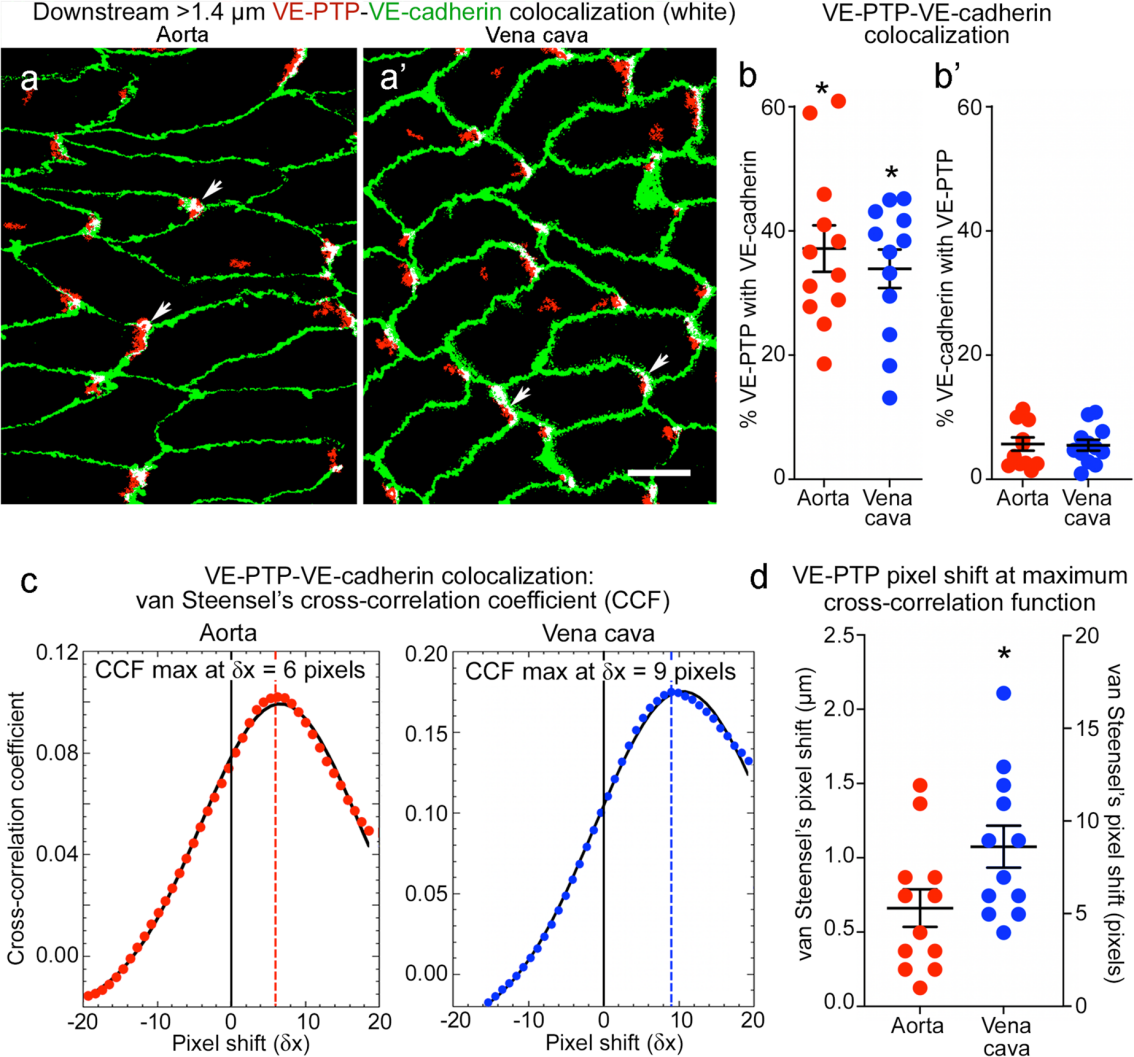
Colocalization of VE-PTP with VE-cadherin in aorta and vena cava. **a**–**a’** Confocal microscopic images of aorta (**a**) and vena cava (**a’**) showing partial colocalization (white) of the largest VE-PTP particles (> 1.4-µm diameter, red) with VE-cadherin (green) at the downstream tip of endothelial cells (arrows). Blood flow left to right. Scale bar: 10 µm. **b**–**b’** Dot plots for aorta (**b**) and vena cava (**b’**) comparing percent of the largest VE-PTP particles (> 1.4 µm) colocalized with VE-cadherin (% of VE-PTP, left dot plots) and percent of VE-cadherin colocalized with VE-PTP (% of VE-cadherin, right dot plots). The percent of VE-PTP colocalized with VE-cadherin was significantly greater (asterisks) than VE-cadherin colocalized with VE-PTP, as expected for colocalization limited to focal regions of plasma membrane. *P < 0.05 by one-way ANOVA followed by Tukey’s multiple comparison test. Mean ± SEM, *n* = 12 images from 6 mice/group. **c** Plots of van Steensel’s peak cross-correlation coefficient (CCF) (ImageJ/Fiji > JACoP plugin) in an aorta and vena cava showing positive pixel shift values at maximal colocalization, indicating that the VE-PTP image was shifted to the right (toward the downstream plasma membrane) at peak CCF. This feature is evidence that large VE-PTP particles at the cell tip in (**a**, **a’**) were composed to two parts, non-colocalized red pixels in the cytoplasm and colocalized white pixels in the plasma membrane. **d** Heterogeneity of pixel shift values for 12 image pairs having peak CCF values with a right shift averaging +5.3 pixels (0.66 µm) in aorta and +8.7 pixels (1.07 µm) in vena cava. Y-axes show CCF values scaled in micrometers (left) and pixels (right). *P < 0.05 by Student’s *t* test. Mean ± SEM, *n* = 12 images from 6 mice/group

Association of the largest VE-PTP particles (diameter > 1.4 µm) with the downstream plasma membrane was evaluated by measuring VE-PTP colocalization with VE-cadherin in both vessels (Fig. 4a, a’). On average, 37% of VE-PTP particles colocalized with VE-cadherin in the aorta and 34% in the vena cava, although individual values varied greatly (Fig. 4b, coefficients of variation 35% and 32%). By comparison, only 5.7% of VE-cadherin colocalized with VE-PTP in the aorta and 5.5% in the vena cava (Fig. 4b’), reflective of VE-PTP particles concentrated in focal regions of the downstream plasma membrane.

Regions of VE-PTP/VE-cadherin colocalization in the plasma membrane were always next to sites of non-colocalized VE-PTP (Fig. 4a, a’). This feature was quantified by plotting van Steensel's Cross-correlation Coefficient (CCF, see Methods) (Fig. 4c). CCF was maximal when the VE-PTP channel was shifted downstream (to the right) in relation to the VE-cadherin channel by +5.3 pixels (0.66 µm) in the aorta and +8.7 pixels (1.07 µm) in the vena cava (Fig. 4c, d). Pixel shift values fit with images of colocalized VE-PTP in the plasma membrane upstream to non-colocalized VE-PTP in the cytoplasm (Fig. 4a, a’). The findings also fit with evidence for two populations of VE-PTP, one in the plasma membrane and the other in the cytoplasm (Mantilidewi et al. 2014; Shirakura et al. 2023).

### Heterogeneity of VE-PTP in the plasma membrane and cytoplasm of endothelial cells

**Fig. 5:**
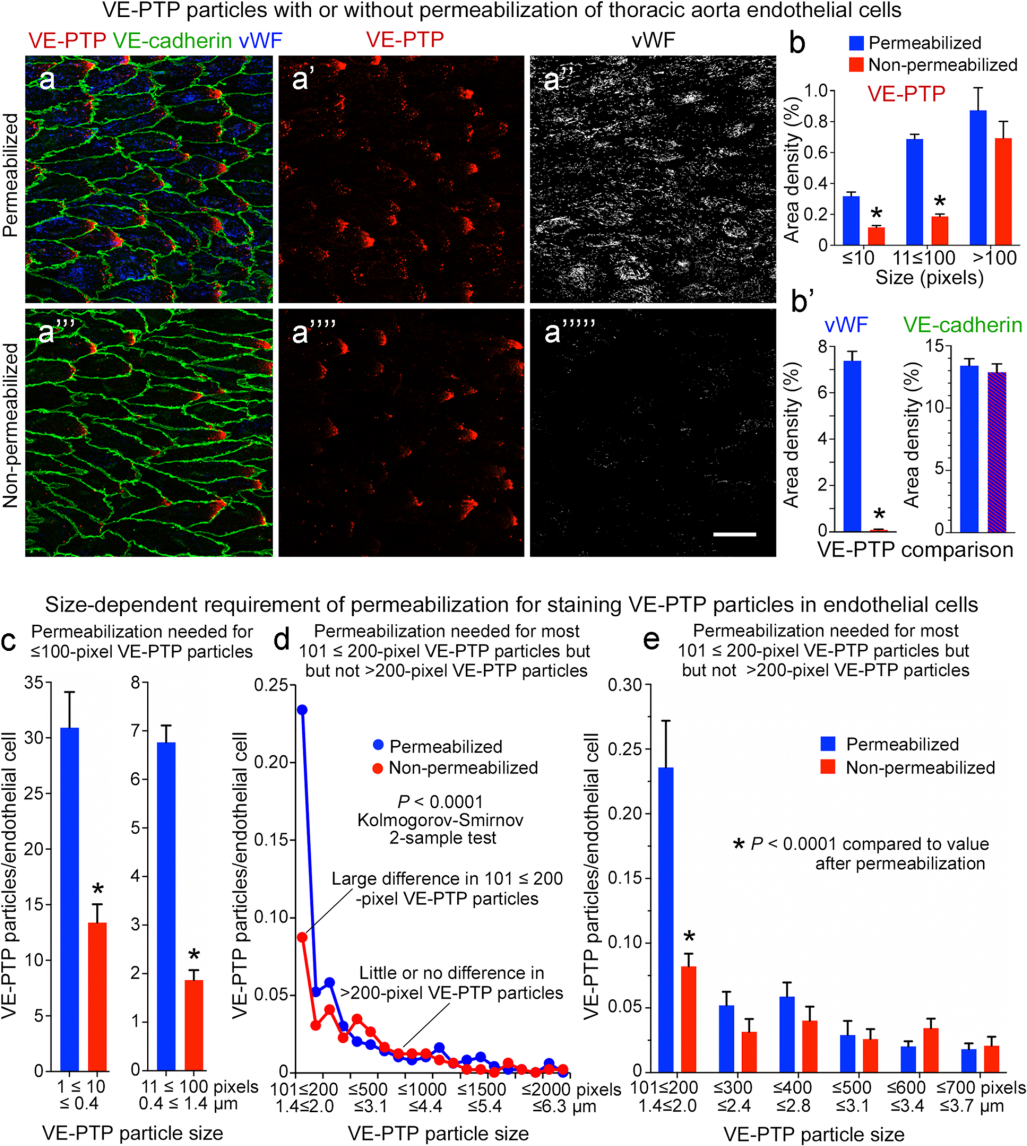
Number and size of VE-PTP particles in permeabilized and non-permeabilized aortic endothelial cells. **a**–**a’’’’’** Confocal microscopic images of VE-PTP (red), Willebrand factor (vWF, blue or white), and VE-cadherin (green) in thoracic aorta endothelial cells with (**a**–**a’’**) or without (**a’’’**–**a’’’’**) permeabilization during staining for VE-PTP and vWF. VE-cadherin was stained in the presence of TritonX-100 in all specimens. Permeabilization was required for vWF staining in cytoplasmic organelles. Most small VE-PTP particles required permeabilization for staining, but the largest particles did not. Scale bar: 25 µm. **b** Area density measurements revealed significantly fewer ≤ 100-pixel VE-PTP particles but similar numbers of larger VE-PTP particles in aortas without permeabilization. **b’** As expected, vWF staining required permeabilization, as almost none was found without TritonX-100. VE-cadherin values were similar in the two groups because TritonX-100 was used for VE-cadherin staining in all specimens (blue/red hashed bar). *P < 0.0001 by ANOVA or Student’s *t* test. **c** Measurements showed significantly fewer ≤ 10-pixel and 11 ≤ 100-pixel VE-PTP particles per endothelial cell without permeabilization. *P < 0.0001 by Student’s *t* test. **d** Line plots of aortas show significantly fewer 101 ≤ 200-pixel VE-PTP particles without permeabilization but similar numbers of > 200-pixel particles, consistent with a cytoplasmic location of most smaller VE-PTP particles and plasma membrane location of larger VE-PTP particles. *P* < 0.0001 by Kolmogorov-Smirnov 2-sample test. **e** Comparison of large VE-PTP particles shows significantly fewer 101 ≤ 200-pixel particles without permeabilization. Permeabilization had little effect on VE-PTP particles > 200 pixels, which fit with a plasma membrane location. *P < 0.0001 by two-way ANOVA. Mean ± SEM, *n* = 12 images from 6 mice/group

Some VE-PTP colocalized with VE-cadherin in images of endothelial cells in 3-dimensional whole mounts could represent VE-PTP in the cytoplasm above or below VE-cadherin in the plasma membrane (Figs. 2a’’, a’’’ and 4a, a’). To circumvent this issue, we also determined the location of VE-PTP by comparing aortas with or without permeabilization during staining (Fig. 5a–a’’’’’). As a reference for the amount of intracellular staining of VE-PTP (Fig. 5b), we measured the intracellular protein vWF, which revealed 90-fold as much vWF staining after permeabilization compared to no permeabilization (Fig. 5b’). As expected, the amount of VE-cadherin was the same under both conditions (Fig. 5b’), because all specimens were stained for VE-cadherin in the presence of TritonX-100 (see Methods). The approach made it possible to distinguish VE-PTP in the cytoplasm (Fig. 5a–a’’, permeabilization required) from VE-PTP in the plasma membrane (Fig. 5a’’’–a’’’’’, permeabilization not required). Area density measurements of VE-PTP staining in non-permeabilized aortas revealed that only 27% of 11 ≤ 100-pixel particles and 36% of ≤ 10-pixel particles were in the plasma membrane, but 79% of > 100-pixel particles were in the plasma membrane (Fig. 5b). Similar results were obtained for ≤ 100-pixel VE-PTP particles when the values were expressed per endothelial cell (Fig. 5c). However, analysis of subsets of > 100-pixel particles revealed that 90% of > 200-pixel particles were in the plasma membrane, compared to only 37% of 101 ≤ 200-pixel particles (Fig. 5d, e). Together, these findings indicate that most ≤ 200-pixel VE-PTP particles were in the cytoplasm, and most > 200-pixel particles were in the plasma membrane, specifically at the downstream tip of endothelial cells.

### Heterogeneity of Tie2-pY992 in endothelial cells of aorta and vena cava

**Fig. 6:**
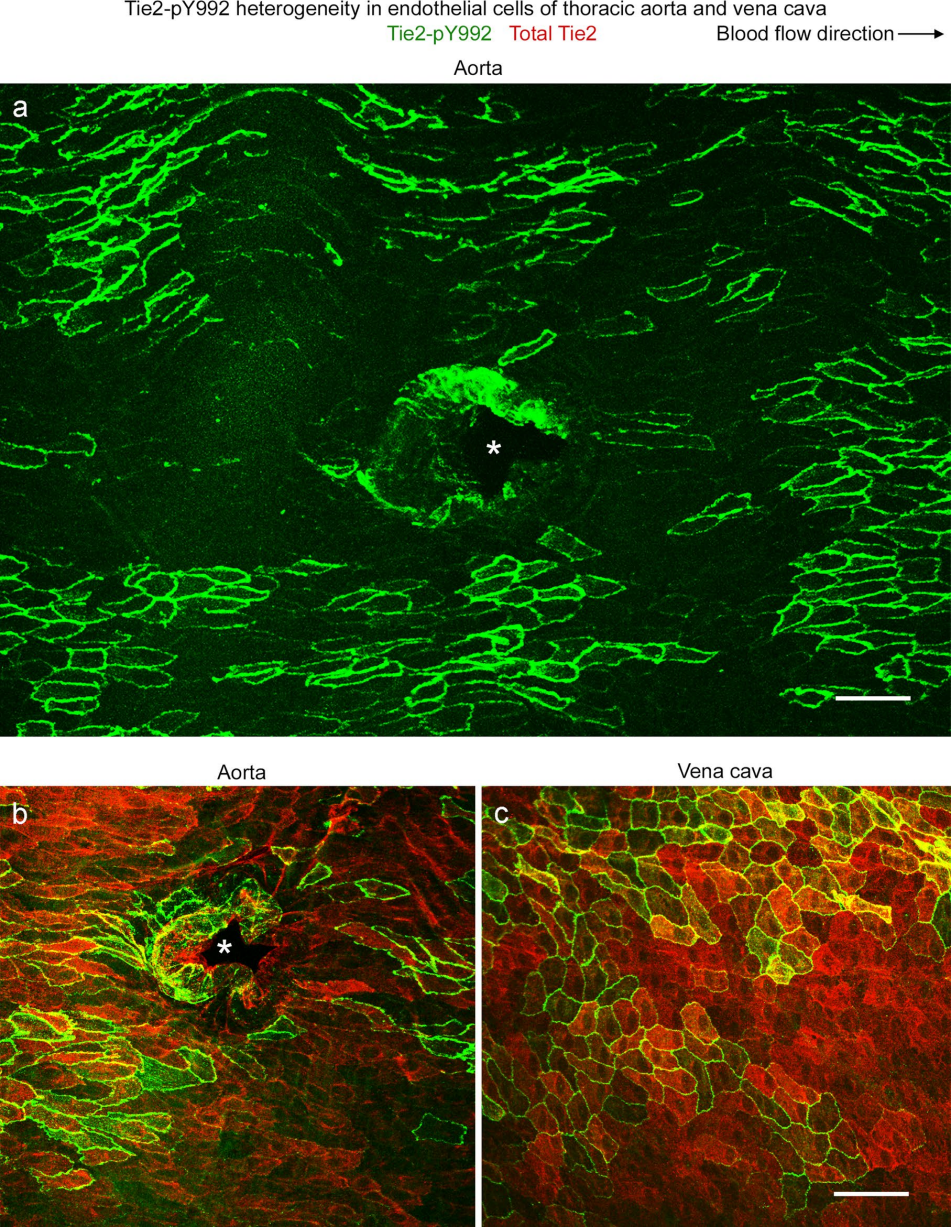
Tie2-pY992 heterogeneity in aorta and vena cava. **a**–**c** Confocal microscopic images showing the heterogeneous distribution of Tie2-pY922 (green) in endothelial cells of the thoracic aorta and vena cava of mice. The mosaic pattern of Tie2-pY992 consists of clusters of endothelial cells with strong staining surrounded by endothelial cells with little or no staining (**a**). Blood flow is left to right. **b**,** c** Broader distribution of overall Tie2 protein (red) than Tie2-pY992 (green) in the aorta (**b**) and vena cava (**c**). In both vessels, Tie2-pY992 staining is strongest at endothelial cell borders, whereas overall Tie2 is widespread. Asterisks mark intercostal artery ostia (**a**,** b**). Endothelial cells of the aorta are more elongated than those of the vena cava. Scale bars: 50 µm

Tie2-pY992 in endothelial cells of the descending thoracic aorta had a mosaic pattern, where clusters of endothelial cells with strong Tie2-pY922 staining were surrounded by endothelial cells with little or none (Fig. 6a). The mosaic of Tie2-pY992 contrasted with the widespread distribution of total Tie2 protein (Fig. 6b). The vena cava had a similar mosaic pattern of Tie2-pY992 and uniform distribution of total Tie2 protein (Fig. 6c). In both vessels, Tie2-pY992 staining was concentrated at the borders and focal adhesions of endothelial cells, but total Tie2 protein was not (Fig. 6b, c).

**Fig. 7:**
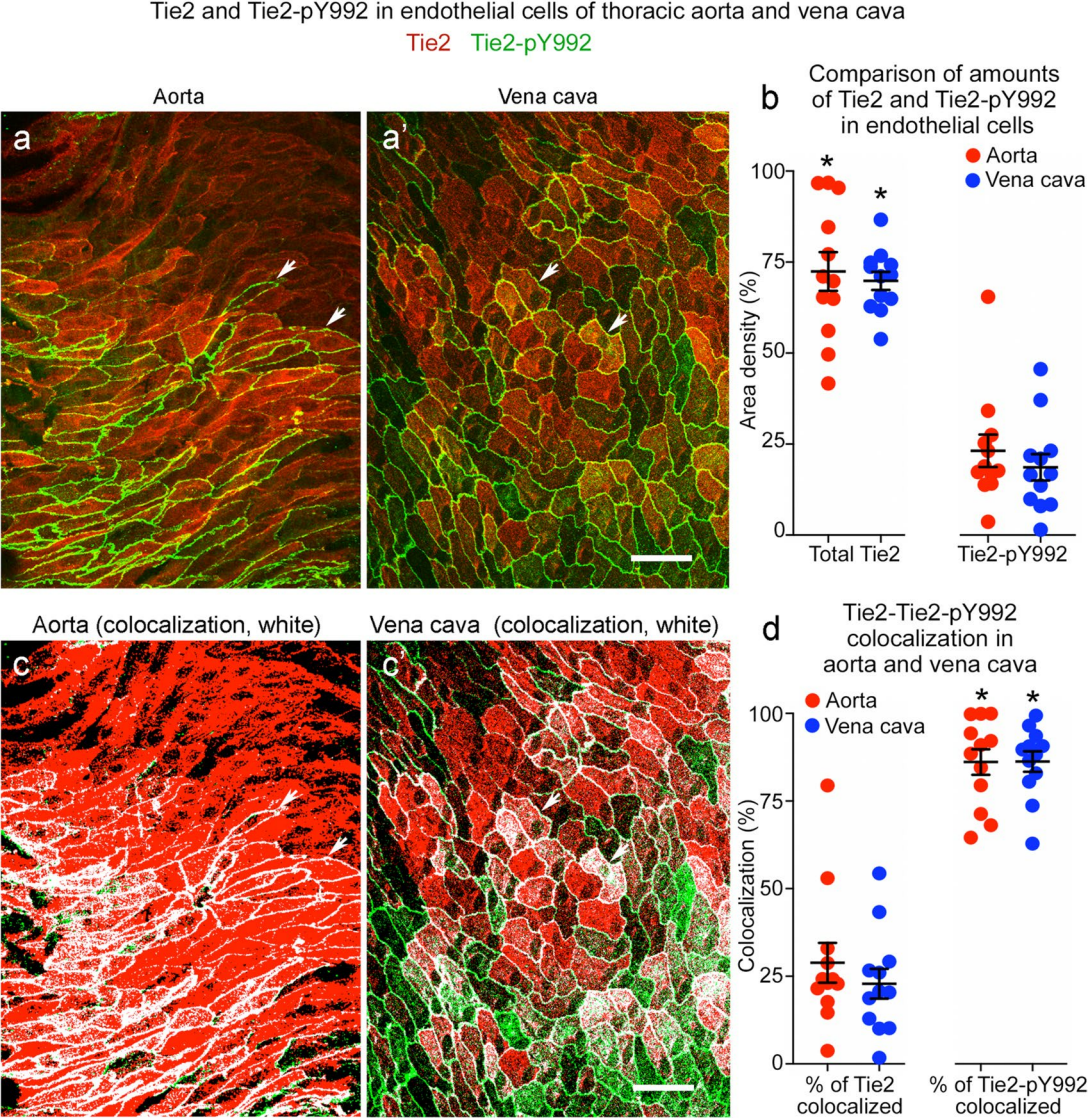
Tie2-pY992 has a restricted distribution in relation to Tie2 in aorta and vena cava. **a**, **a’** Confocal microscopic images of total Tie2 (red) and Tie2-pY922 (green) staining in endothelial cells of the descending thoracic aorta (**a**) and vena cava (**a’**). Tie2 is much more widespread than Tie2-pY992, which is restricted to a subset of endothelial cells. Scale bar: 20 µm. **b** Measurements documenting the broader distribution of Tie2 than Tie2-pY992 and similarity of amounts of both in the aorta and vena cava. **c**, **c’** The same images as in (**a**, **a’**), here showing the distribution of Tie2-pY992/Tie2 colocalization (white) in the aorta (**c**) and vena cava (**c’**). Scale bar: 20 µm. **d** Measurements of Tie2-pY992/Tie2 colocalization expressed as the percent of Tie2 colocalized (left dot plots) and percent of Tie2-pY992 colocalized (right dot plots). The plots show that 86% of Tie2-pY992 colocalized with Tie2 (right), but only 29% of Tie2 in aorta and 23% of Tie2 in vena cava colocalized with Tie2-pY992 (left). *P < 0.05 by one-way ANOVA followed by Tukey’s multiple comparison test. Mean ± SEM, *n* = 12 images from 6 mice/group

Area density measurements revealed that Tie2-pY992 staining represented about 25% of the area of total Tie2 protein in the aorta and vena cava (Fig. 7a, a’, b). Tie2-pY992 was much more variable from region to region than total Tie2. The coefficient of variation for Tie2-pY992 was 66% in the aorta and 67% in the vena cava, compared to 25% and 12% for total Tie2. As expected, most Tie2-pY992 colocalized with total Tie2, but on average, only 25% of Tie2 colocalized with Tie2-pY992 (Fig. 7c, c’, d).

**Fig. 8:**
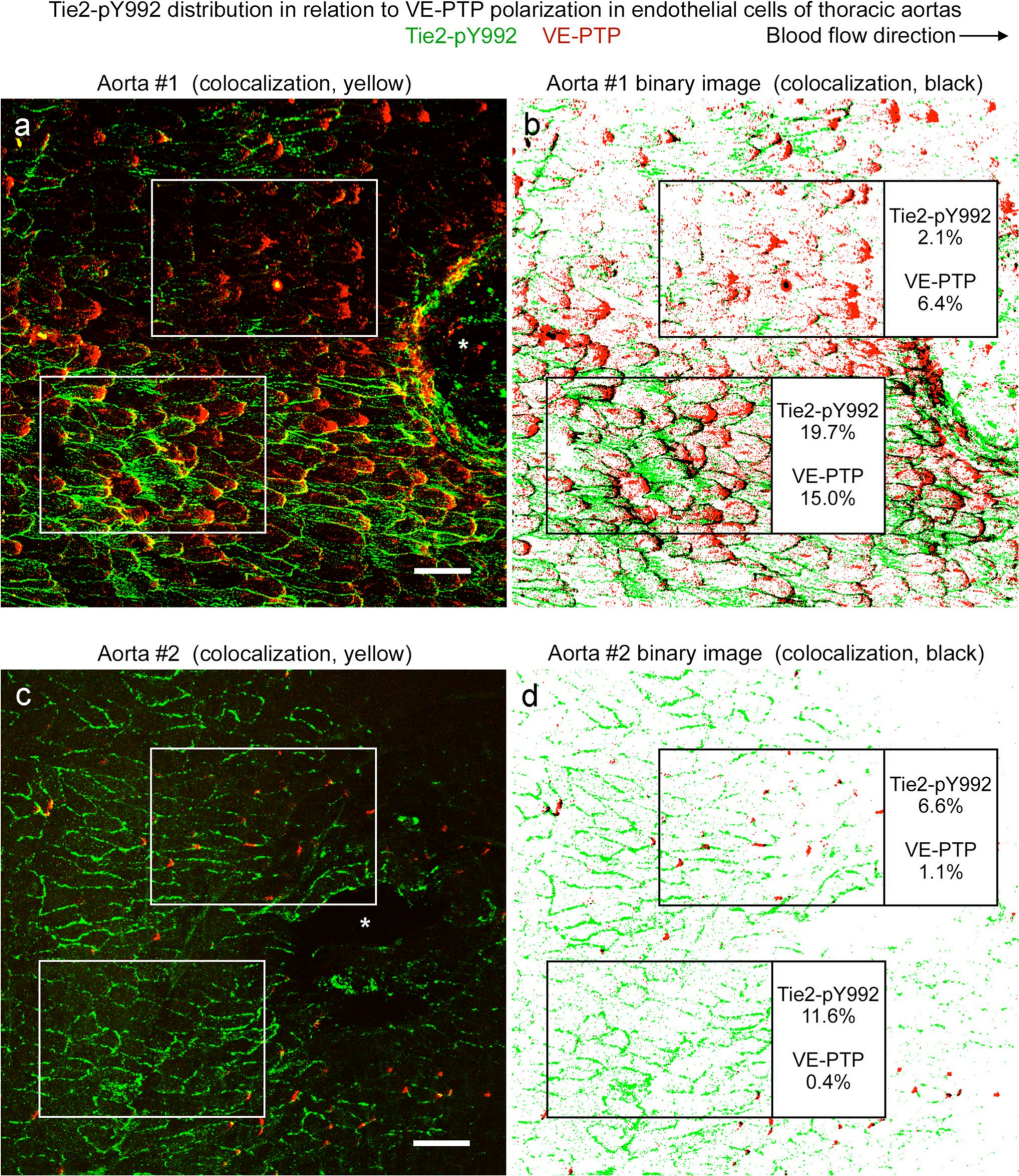
Tie2-pY992 distribution in relation to VE-PTP polarization in aorta. **a**–**d** Confocal microscopic images (**a**, **c**) of endothelial cells in descending thoracic aorta upstream to intercostal artery ostia (asterisks) and corresponding color-coded binary images (**b**, **d**) comparing heterogeneous amounts of Tie2-pY992 staining (green) and VE-PTP polarization (red) in two aortas. Blood flow left to right. Measured fractional areas of Tie2-pY992 and VE-PTP are shown in white boxes (area density, %). **a**,** b** Aorta #1: Two regions of endothelium (boxes) with weak Tie2-pY992 and moderate VE-PTP polarization in the upper box and strong Tie2-pY992 and strong VE-PTP polarization in the lower box. **c**,** d** Aorta #2: Adjacent regions (boxes), both having moderate Tie2-pY992 and weak VE-PTP polarization in the endothelium of another aorta. Scale bars: 50 µm

**Fig. 9:**
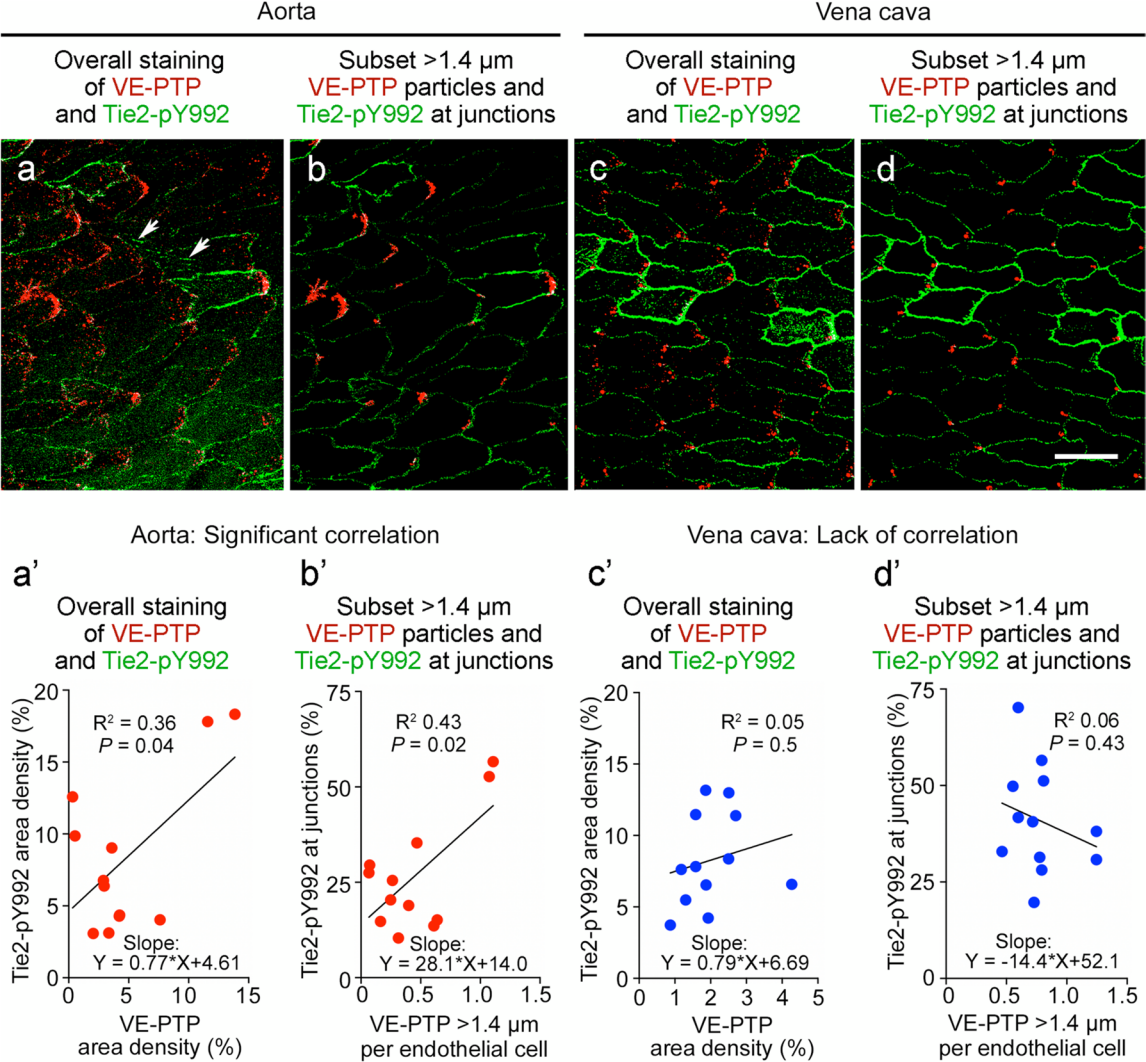
Tie2-pY992 and VE-PTP correlation in endothelial cells of aorta but not vena cava. Confocal microscopic images (**a**–**d**) and linear regression plots (**a’**–**d’**) of Tie2-pY992 (green) and VE-PTP (red) in endothelial cells of thoracic aorta (**a**, **a**’, **b**, **b’**) and vena cava (**c**, **c’**, **d**, **d’**). **a** Image of aorta showing heterogeneity of downstream polarization of VE-PTP particles and Tie2-pY992 at intercellular junctions and focal adhesions (arrows). **a’** Regression plot showing significant correlation of Tie2-pY992 and VE-PTP area densities. *P* = 0.04. **b** Same image as in (**a**) here showing only > 1.4 µm VE-PTP particles and Tie2-pY992 at cell junctions. **b’** Regression plot showing significant correlation between junctional Tie2-pY992 and number of > 1.4 µm VE-PTP particles. *P* = 0.015, *n* = 12 images from 5 mice. **c** Image of inferior vena cava showing heterogeneous Tie2-pY992 staining and VE-PTP polarization in endothelial cells. **c’** Regression plot documenting the heterogeneity and lack of correlation between Tie2-pY992 and VE-PTP in the vena cava. *P* = 0.5. **d** Same region in (**c**) here showing only > 1.4 µm VE-PTP particles and Tie2-pY992 at cell junctions. **d’** Regression plot showing lack of correlation between junctional Tie2-pY992 and > 1.4 µm VE-PTP particles in the vena cava. *P* = 0.43, *n* = 12 images from 6 mice. Scale bar: 20 µm

Tie2-pY992 and VE-PTP were stained together in the same vessels to determine whether their mosaic patterns coincided. Comparisons revealed that Tie2-pY992 and VE-PTP were similarly strong or weak in some but not all regions of the thoracic aorta (Fig. 8a–d). Despite the heterogeneity evident in confocal microscopic images of both vessel types (Fig. 9a–d), measurements revealed significant correlations in the overall area densities of Tie2-pY992 and VE-PTP and in the amount of Tie2-pY992 at junctions and number of > 1.4-µm VE-PTP particles per cell in the aorta (Fig. 9a’, b’) but not in the vena cava (Fig. 9c’, d’).

### Heterogeneity of claudin-5 in endothelial cells of aorta and vena cava

Transmembrane proteins that form intercellular junctions are essential elements of the endothelial cell barrier (Claesson-Welsh et al. 2021; Dejana et al. 2000). To assess the heterogeneity of junctional proteins in the thoracic aorta and inferior vena cava, we examined claudin-5 at tight junctions and VE-cadherin at adherens junctions (Corada et al. 1999; Dejana and Giampietro 2012; Morita et al. 1999; Richards et al. 2022).

**Fig. 10:**
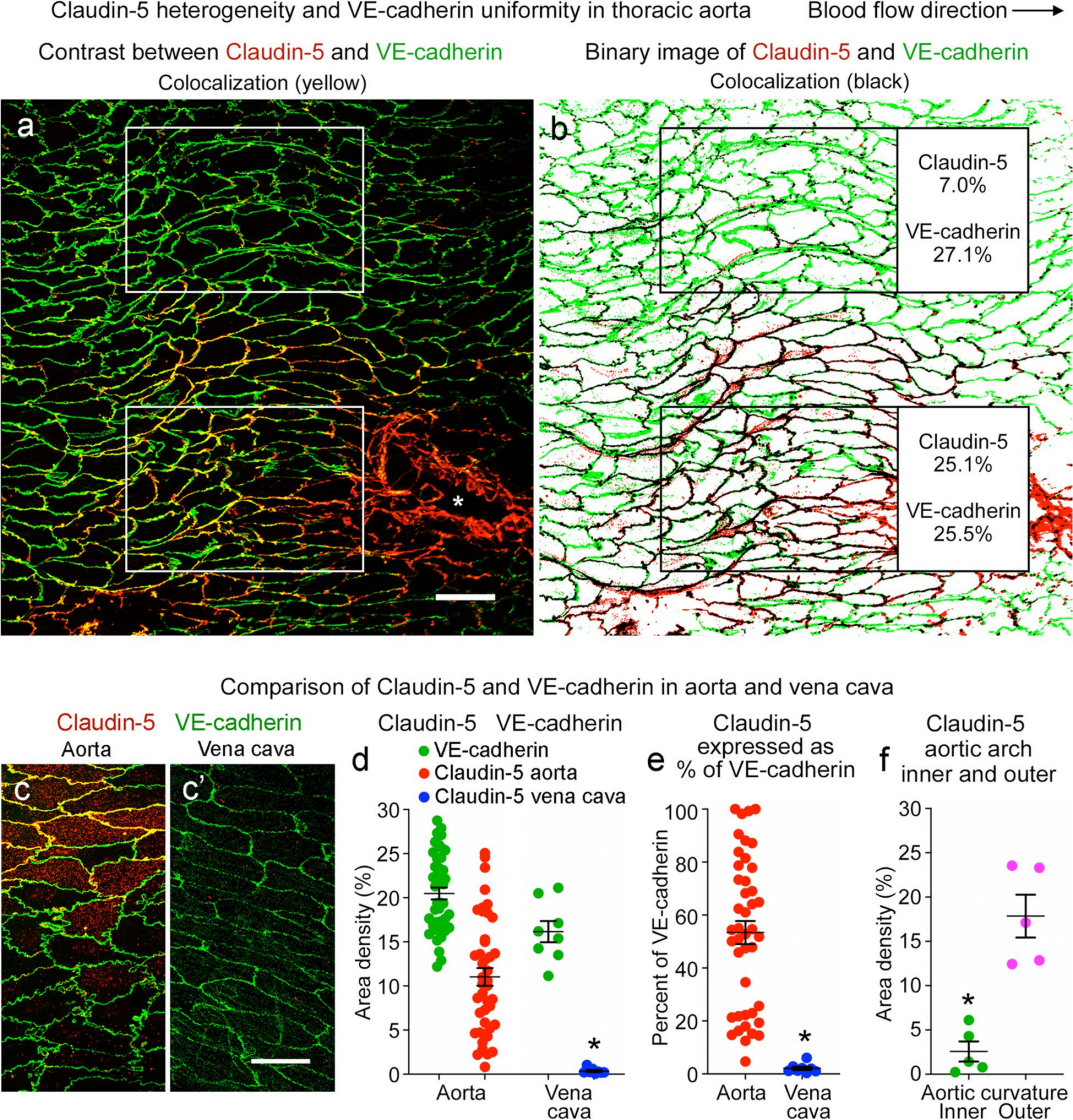
Claudin-5 heterogeneity in aorta and absence in vena cava. **a** Confocal microscopic image of a region of descending aorta upstream to an intercostal artery ostium (asterisk) illustrating the heterogeneity of claudin-5 (red) and the uniform distribution of VE-cadherin (green) in endothelial cells. Blood flow left to right. Scale bar: 50 µm. **b** Color-coded binary version of image (**a**) showing amounts of claudin-5 and VE-cadherin in boxed regions. In the upper box, claudin-5 (area density 7.0%) was only 26% of VE-cadherin (area density 27.1%), but in the lower box claudin-5 (area density 25.1%) was 98% of VE-cadherin (area density 25.5%). **c**, **c’** Confocal microscopic images comparing amounts of claudin-5 (red) and VE-cadherin (green) in thoracic aorta (**c**) and inferior vena cava (**c’**). Claudin-5 staining is patchy in the aorta and absent in the vena cava. Scale bar: 20 µm. **d** Measurements comparing the heterogeneity in amount of claudin-5 and VE-cadherin staining in the aorta and vena cava. **e** Dot plots showing amount of claudin-5 in the aorta and vena cava expressed as percent of VE-cadherin. *P < 0.0001 by Student’s *t* test. Mean ± SEM, *n* = 43 regions of aorta in 10 mice and *n* = 8 regions of vena cava in 5 mice. **f** Area density of claudin-5 in the inner and outer curvatures of aortic arch. *P < 0.001 by Student’s *t* test. Mean ± SEM, *n* = 5 images of each region of aortic arch and 9 images of vena cava in 5 mice

Consistent with previous reports (Engelbrecht et al. 2020; Miao et al. 2005), VE-cadherin uniformly surrounded endothelial cells of the thoracic aorta and vena cava (Fig. 10a, b). However, claudin-5 did not surround all endothelial cells but instead had a mosaic pattern at endothelial cell borders in the aorta (Fig. 10a–c) and was nearly absent in the vena cava (Fig. 10c’). The mean area density of claudin-5 in the aorta (11%) was 31 times the value in the vena cava (0.35%), whereas the area density of VE-cadherin was similar in both vessels (20% and 16%) (Fig. 10d). The heterogeneity of claudin-5 in the aorta was reflected by the coefficient of variation of 60%, compared to 21% for VE-cadherin (Fig. 10d). Consistent with this heterogeneity, claudin-5 varied from 5 to 100% (mean 53 ± 4%) of the amount of VE-cadherin in the thoracic aorta (Fig. 10e), where a value of 100% indicates complete encirclement of endothelial cells. These values for the vena cava ranged from 0.4% to 6% (mean 2.1 ± 0.6%) (Fig. 10e). In the aortic arch, claudin-5 was sparse in the inner curvature (2.6% area density, which was only 9% of VE-cadherin), compared to 18% area density and 59% of VE-cadherin in the outer curvature (Fig. 10f).

**Fig. 11:**
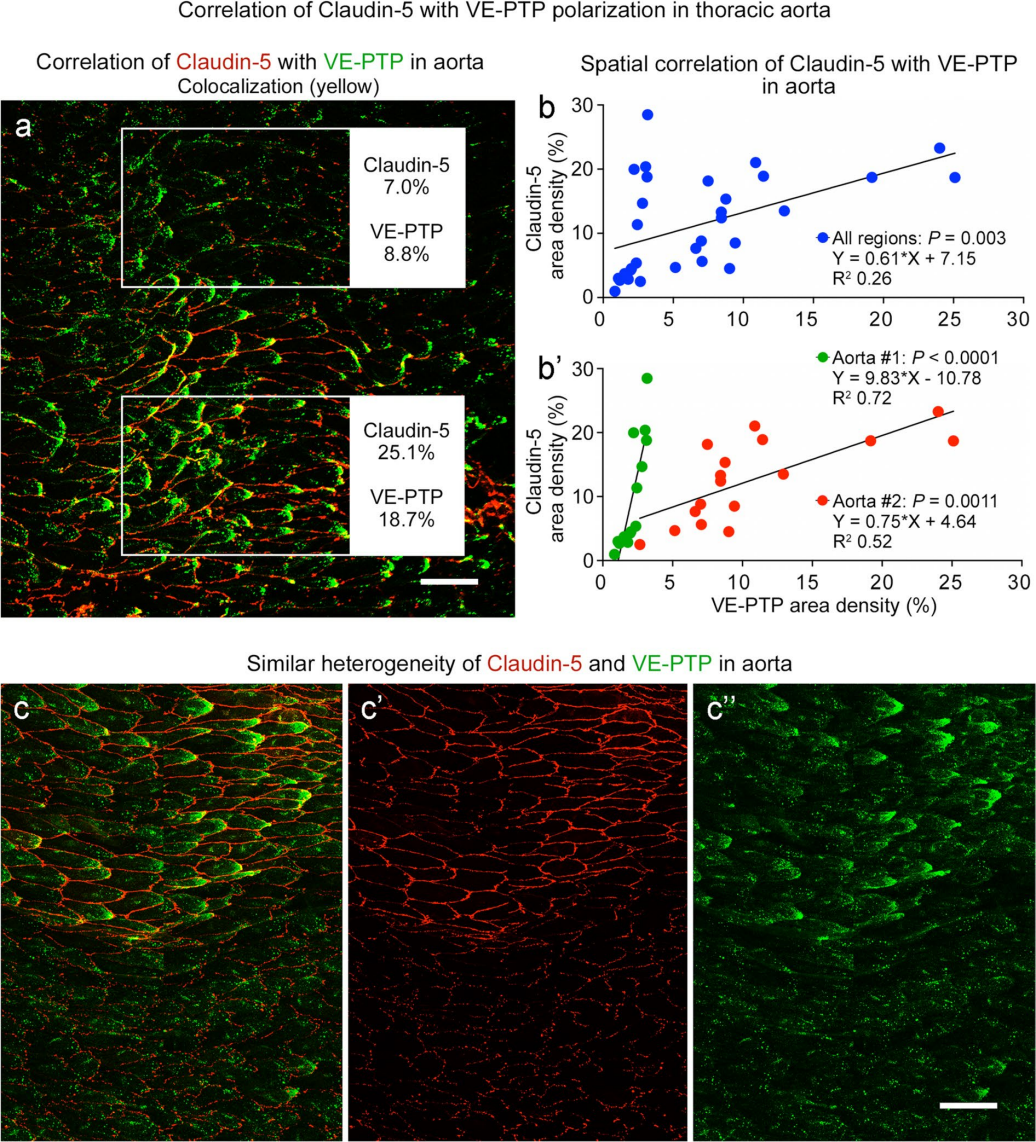
Claudin-5 and VE-PTP correlation in endothelial cells of aorta. **a** Confocal microscopic image of the aortic region shown in Fig. 10a, here comparing claudin-5 (red) and VE-PTP (green) staining. Upper box shows a region with little claudin-5 or VE-PTP. Lower box shows an adjacent region with abundant claudin-5 and VE-PTP. Claudin-5 and VE-PTP area densities are shown in outlined regions. Scale bar: 50 µm. **b**, **b’** Linear regression plots showing significant correlation of claudin-5 and VE-PTP in endothelial cells in two aortas. **b** shows values for 32 regions plotted together (*P* = 0.003). **b’** shows the same values plotted separately for the two aortas. Claudin-5 and VE-PTP are significantly correlated in both curves, but the slopes are different. Aorta #1, *P* < 0.0001, *n* = 15 regions. Aorta #2, *P* = 0.0011, *n* = 17 regions. **c**–**c’’** Confocal microscopic image of claudin-5 (red) and VE-PTP (green) in aorta, shown here together (**c**) and separately (**c’** claudin-5, **c’’** VE-PTP), to illustrate the similar heterogeneities in another aorta (Aorta #5, Fig. 12a’’, a’’’’’) that was sampled around the entire circumference. Scale bar: 50 µm

The question of whether the mosaic of claudin-5 matched the pattern of VE-PTP polarization in co-stained aortas revealed that both were sparse in some regions (Fig. 11a, upper box) and extensive in others (Fig. 11a, lower box). The amounts of claudin-5 and VE-PTP were found to be significantly correlated by linear regression analysis of 32 regions in two aortas (Fig. 11b). Analysis of each aorta separately showed even stronger correlations but different slopes for the two aortas (Fig. 11b’).

**Fig. 12:**
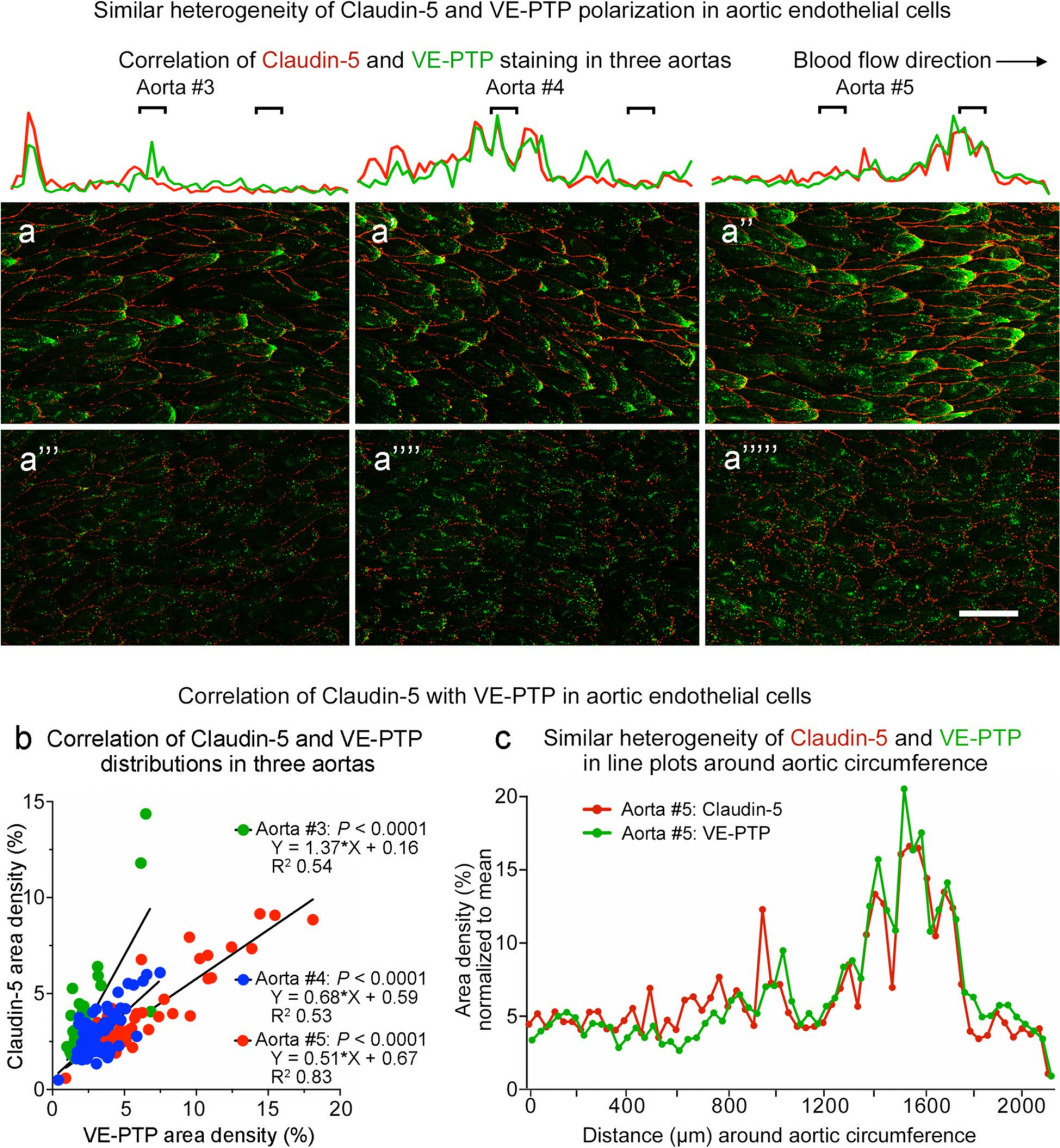
Similar heterogeneity of claudin-5 and VE-PTP around aortic circumference. **a**–**a’’’’’** Confocal microscopic images of claudin-5 (red) and VE-PTP (green) comparing regions with strong (**a**–**a’’**) and weak (**a’’’**–**a’’’’’**) staining in the three aortas numbered #3–5 that were sampled around the entire circumference (values for aortas #1–2 are shown in Fig. 11b, b**’**; aorta #5 is also shown in Fig. 11c–c’’). Blood flow left to right. Line plots above the images show similar heterogeneities of fluorescence intensities of claudin-5 (red) and VE-PTP (green) sampled around the entire 2-mm circumference of each aorta. Black lines mark the locations of corresponding images. Scale bar: 20 µm. **b** Linear regression plots comparing claudin-5 and VE-PTP in endothelial cells around the circumference of the three aortas shown in (**a**–**a’’’’’**). The plots show significant correlation (*P* < 0.0001) of claudin-5 and VE-PTP measured in 61–73 regions (1500 × 250 pixels each) sampled sequentially around each aorta. **c** Line plots showing similarly heterogenous claudin-5 and VE-PTP fluorescence sampled around the circumference of aorta #5 in (**a’’**, **a’’’’’**). Claudin-5 and VE-PTP fluorescence intensities were standardized by normalizing values to the respective mean. No significant difference (*P* > 0.5) by Kolmogorov-Smirnov 2-sample test

The relationship between claudin-5 and VE-PTP was further tested by systematic analysis of continuous bands of endothelium around the entire circumference of aortas stained for both proteins (Fig. 11c). Confocal microscopic imaging confirmed the presence of coincident gradients of staining for claudin-5 (Fig. 11c’) and VE-PTP (Fig. 11c’’). Both were strong or weak in the same regions (Fig. 11c–c’’). Comparison of three aortas in this manner revealed that the distributions of claudin-5 and VE-PTP matched in each aorta but differed from aorta to aorta (Fig. 12a–a’’’’’). Correlation of claudin-5 and VE-PTP was confirmed by linear regression analysis of 61–73 test regions around the circumference of the three aortas (Fig. 12b) and was also evident from the similarity of line plots of claudin-5 and VE-PTP fluorescence around the circumference (Fig. 12c).

### Heterogeneity of extravasated IgG in aorta and vena cava

**Fig. 13:**
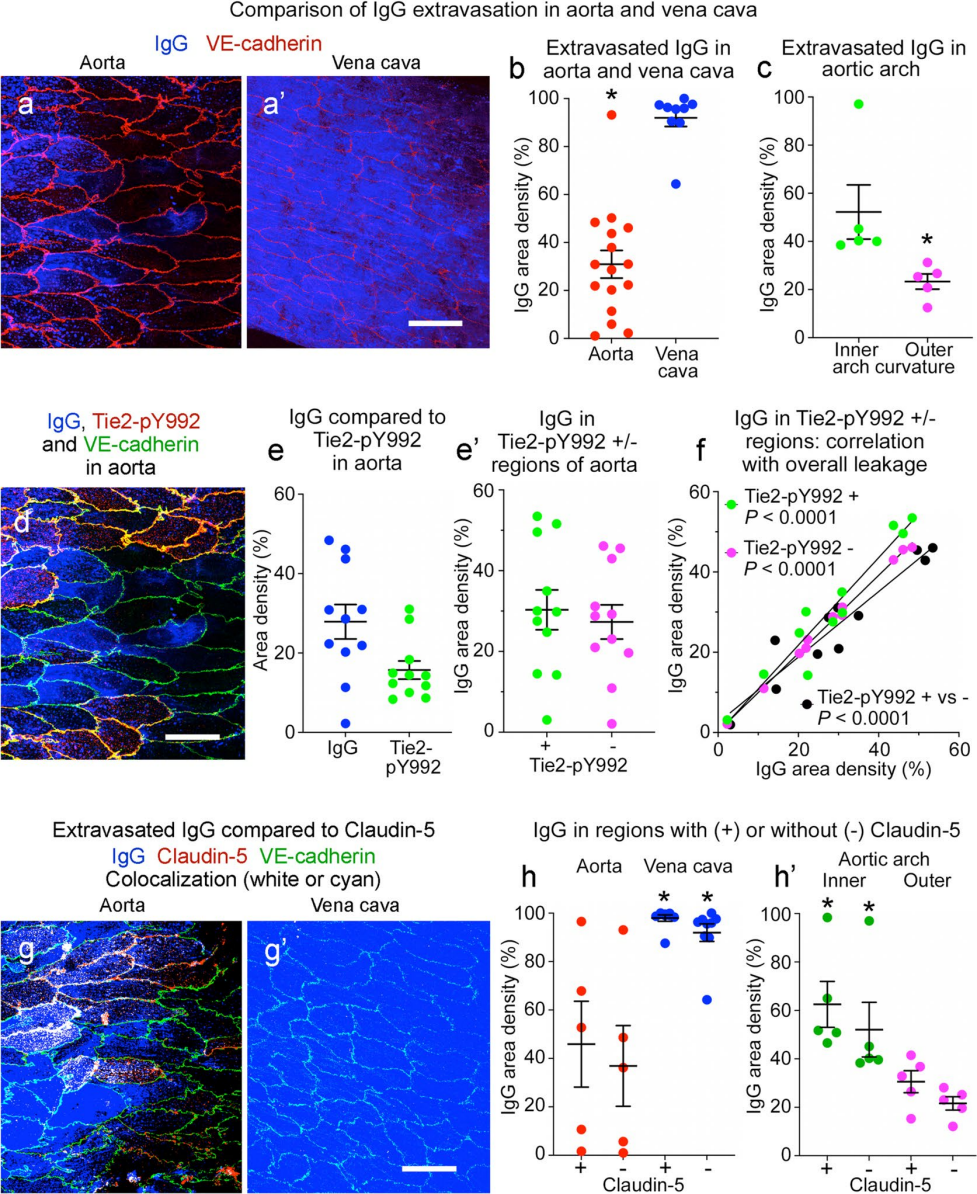
Heterogeneity of extravasated IgG in aorta and vena cava. **a**,** a’** Confocal microscopic images showing patchy extravasated IgG (blue) in thoracic aorta (**a**) and widespread IgG in vena cava (**a’**). VE-cadherin (red). **b** Heterogeneity of IgG in aorta (mean 31%) and vena cava (mean 92%). *n* = 16 and 9 images from 6 mice, *P < 0.001 by Student’s *t* test. **c** Heterogeneity of IgG in aortic arch inner (mean 52%) and outer curvature (mean 23%). *n* = 5 images of each from 5 mice, *P < 0.05 by Student’s *t* test. **d** Aorta in (**a**) here showing IgG (blue) in relation to Tie2-pY992 (red) and VE-cadherin (yellow/green). **e**,** e’** Heterogeneity of IgG and Tie2-pY992 in aorta (**e**) and similar amounts of IgG in regions with or without Tie2-pY992 (**e’**). *n* = 11 images from 6 mice. *P* = 0.65 by Student’s *t* test. **f** Linear regression plots showing strong correlation (*P* < 0.0001) between IgG in regions of aorta with or without Tie2-pY992 (+ vs -, black) and between IgG in regions with (+, green) or without Tie2-pY992 (-, magenta) and total IgG. *n* = 11 images from 6 mice. Tie2-pY992-positive regions: R^2^ 0.925, *P* < 0.0001. Tie2-pY992-negative regions: R^2^ 0.997, *P* < 0.0001. IgG in Tie2-pY992-positive vs. -negative: R^2^ 0.903, *P* < 0.0001. **g**, **g’** Images contrasting patchy claudin-5 (red) and IgG (blue) in aorta (**g**) with no claudin-5 and widespread IgG in vena cava (**g’**). VE-cadherin (green). **h**,** h’** Heterogeneous but roughly equal amounts of IgG in regions with or without claudin-5 in thoracic aorta and vena cava (**h**) and aortic arch inner and outer curvatures (**h’**). *P* < 0.001 by Student’s *t* test. *n* = 5 images in thoracic aorta and aortic arch, 9 images in vena cava from 5 mice**.** Graphs show individual values and mean ± SEM expressed as percent area density. Scale bars: 20 µm

Regions of the aorta exposed to turbulent flow are predisposed to leakage and development of atherosclerotic plaques (Davies 1995; Gimbrone and Garcia-Cardena 2013). The inner curvature of the aortic arch, which has little VE-PTP polarization and weak Tie2 activation in endothelial cells, is among such regions (Shirakura et al. 2023). The present studies confirmed the presence of scattered regions of extravasated IgG in the descending thoracic aorta (Fig. 13a) and much more widespread IgG in the vena cava (Fig. 13a’). On average, 31% of 16 regions of aorta sampled had extravasated IgG, but the range was 1 to 93% (coefficient of variation 75%) (Fig. 13b). By comparison, 92% of the inferior vena cava had IgG with little variability (coefficient of variation 12%) (Fig. 13b). Corresponding mean values for IgG accumulations in the aortic arch were 23% in the outer curvature and 52% in the inner curvature (Fig. 13c).

The heterogeneity of IgG extravasation in the descending thoracic aorta raised the question of whether leakage was greater in regions with less Tie2-pY992 or claudin-5. The contribution of Tie2-pY992 was addressed by comparing IgG accumulations in regions with or without Tie2-pY992 staining (Fig. 13d). Measurements revealed that staining for IgG was more variable than for Tie2-pY992 (Fig. 13e) but on average was similar in regions with or without Tie2-pY992 staining (Fig. 13e’). Consistent with this similarity, IgG in regions with or without Tie2-pY992 correlated with each other and with the overall amount of leakage (R^2^ > 0.9, Fig. 13f). IgG accumulations were also similar in regions with or without claudin-5 in the thoracic aorta (Fig. 13g, h), vena cava (Fig. 13g’, h), and aortic arch inner and outer curvatures (Fig. 13h’).

## Discussion

This study characterized the heterogeneity of VE-PTP, Tie2-pY992, and claudin-5 in endothelial cells of the aorta and vena cava. The work built on evidence that laminar blood flow and high shear stress promote downstream polarization of VE-PTP, increased Tie2-pY992, and reduced endothelial cell permeability (Shirakura et al. 2023). We found striking heterogeneity of staining for VE-PTP, Tie2-pY992, and claudin-5 in endothelial cells of anatomically defined regions of the descending thoracic aorta. VE-PTP appeared as particles ranging in size from the threshold of detection by confocal microscopy (~ 0.1 µm) to patches larger than 2 µm in diameter. Permeabilization studies revealed that most VE-PTP particles smaller than 2 µm were in the cytoplasm, whereas most larger than 2 µm were in the plasma membrane at the downstream pole of endothelial cells. Tie2-pY922 had a mosaic pattern that represented only 25% of total Tie2 staining and correlated with the amount of VE-PTP polarization. Claudin-5 at tight junctions also had a mosaic pattern that correlated with VE-PTP polarization, whereas VE-cadherin at adherens junctions uniformly bordered all endothelial cells. In the vena cava, VE-PTP polarization and Tie2-pY992 had mosaic patterns as in the aorta, but claudin-5 was nearly absent. Focal accumulations of extravasated IgG had a patchy distribution in the wall of the thoracic aorta but were widespread in the vena cava. These heterogeneities are consistent with the local dynamics of blood flow and shear stress and add to previously identified differences among endothelial cells in the aorta and vena cava.

### Approach developed to assess endothelial cell heterogeneity

Heterogeneities in VE-PTP, Tie2-pY992, and claudin-5 were identified by analyzing endothelial cells in immunohistochemically stained 3-dimensional whole mounts of descending thoracic aorta and inferior vena cava (Shirakura et al. 2023). The variability of these features was quantified in confocal microscopic images of anatomically defined regions of the vessels prepared at standardized magnifications and microscope settings. These procedures combined with dual staining by pairs of antibodies revealed previously unrecognized coincident mosaic distributions of VE-PTP polarization, Tie2-pY992, and claudin-5. Although the relationship of VE-PTP to Tie2-pY992 and claudin-5 was measured in the same endothelial cells, corresponding correlations between Tie2-pY992 and claudin-5 were not tested because both antibodies were made in the same species.

Assessment of plasma leakage by measuring IgG in the aortic wall has the attribute of exploiting strong fluorescence signals from historical accumulations of extravasated IgG but proved to have a limitation. The lack of IgG correlation with Tie2-pY992 or claudin-5 did not fit with expectations based on our previous study of the mouse aortic arch, where amounts of extravasated anti-fibrinogen antibody or rabbit IgG circulating for 30 min had an inverse relationship with VE-PTP polarization and Tie2-pY992 (Shirakura et al. 2023). However, the seemingly paradoxical finding of no such relationship in the present study could be explained by (i) spreading of endogenous IgG away from sites of leakage over prolonged periods of accumulation within the vessel wall, and (ii) different half-lives of IgG accumulation/clearance compared to VE-PTP, Tie2-pY992, or claudin-5 dynamics in the thoracic aorta. VE-PTP polarization and Tie2 phosphorylation can change in minutes (Lee and Koh 2003; Shirakura et al. 2023; Teichert-Kuliszewska et al. 2001), whereas the half-life of IgG accumulations in the aorta is governed by the rate of clearance from tissue, which is more than 24 h (Magnussen et al. 2005). When technically feasible, the issue deserves reexamination by comparing the rapid kinetics of VE-PTP, Tie2-pY992, and claudin-5 to extravasation of a tracer that can be measured in real-time.

### Heterogeneity of endothelial cell VE-PTP polarization

VE-PTP is polarized downstream in endothelial cells in regions of thoracic aorta and outer arch curvature exposed to high shear stress but not where flow is disturbed and shear stress is low (Shirakura et al. 2023). Similarly, VE-PTP becomes polarized in cultured human umbilical vein endothelial cells (HUVEC) exposed to high shear stress for 5 min but not under static conditions (Shirakura et al. 2023). VE-PTP redistribution in HUVEC exposed to laminar flow and in the aorta and vena cava match the VE-PTP polarization first identified in cultured bEnd.3 mouse endothelioma cells (Mantilidewi et al. 2014).

The present study extends these findings by documenting the heterogeneity of VE-PTP polarization in anatomically defined regions of the thoracic aorta and vena cava. In the thoracic aorta, VE-PTP polarization was conspicuous in endothelial cells around some intercostal artery ostia. The findings fit with functional evidence of blood flow and shear stress dynamics in these regions (Cheng et al. 2003; Joseph et al. 2020; Mohamied et al. 2017) and expand the parts of the vasculature where VE-PTP is concentrated in the downstream pole of endothelial cells.

Comparison of aortas with or without permeabilization during staining revealed two populations of VE-PTP. VE-PTP in the plasma membrane stained without permeabilization, whereas VE-PTP staining in the cytoplasm required permeabilization similar to vWF. This approach revealed that 90% of VE-PTP particles > 2 µm in diameter (> 200 pixels) were in the plasma membrane, specifically at the downstream tip of endothelial cells. The remaining 10% were in the cytoplasm near the plasma membrane, consistent with VE-PTP in tightly packed clusters of endosomes. These findings fit with evidence from CCF plots of VE-PTP colocalization with VE-cadherin in these regions. Unlike larger VE-PTP particles, 73% of VE-PTP particles 0.4 ≤ 1.4 µm in diameter (11 ≤ 100 pixels) were located in the cytoplasm, mainly in the downstream half of endothelial cells, and resembled endosomes that express Early endosome antigen 1 (EEA1) or Ras-associated protein Rab5 (Rab5) (Shirakura et al. 2023). The smallest VE-PTP particles (≤ 0.4 µm) were more uniformly distributed in endothelial cells.

### Tie2-pY992 heterogeneity and correlation with VE-PTP polarization

VE-PTP is a plasma membrane phosphatase that contributes to the regulation of endothelial barrier function through actions on Tie2 receptors (Campochiaro et al. 2015; Dominguez et al. 2007; Drexler et al. 2019; Fachinger et al. 1999; Frye et al. 2015; Shen et al. 2014; Winderlich et al. 2009). VE-PTP actions on VE-cadherin, VEGFR2, endothelial nitric oxide synthase (eNOS), and the GTPase exchange factor FGD5 can also participate in barrier regulation (Braun et al. 2019; Broermann et al. 2011; Frye et al. 2015; Hayashi et al. 2013; Mellberg et al. 2009; Nawroth et al. 2002; Nottebaum et al. 2008; Siragusa et al. 2021).

Tie2 phosphorylation at tyrosine 992 (Tie2-pY992) promotes endothelial barrier tightening, and dephosphorylation leads to barrier disruption and leakage (Kim et al. 2016; Shirakura et al. 2023). The mosaic pattern of Tie2-pY992 in endothelial cells identified in the present study fits with the known variability of shear stress in the descending aorta and with the blood flow dynamics in the vena cava, where flow is pulsatile and has mixed cardiac and respiratory patterns (Cheng et al. 2003; Joseph et al. 2020; Mohamied et al. 2017).

Correlation of Tie2-pY992 staining with VE-PTP polarization is consistent with stronger Tie2-pY992 in the aortic arch outer curvature, where VE-PTP polarization is prominent and the barrier is tighter, than in the inner curvature, where VE-PTP is not polarized and the barrier is leakier (Shirakura et al. 2023). Similarly, both Tie2 activation and VE-PTP are required for laminar flow suppression of leakage in HUVEC, as shown by siRNA knockdown (Shirakura et al. 2023). Yet, additional studies of VE-PTP polarization, Tie2 phosphorylation, and blood flow dynamics are needed for a complete understanding of their interaction in the regulation of endothelial barrier function in the aorta and vena cava.

### Claudin-5 heterogeneity and correlation with VE-PTP polarization

Claudin-5, other claudins, occludin, ESAM, JAM-A, and related proteins at tight junctions, together with VE-cadherin at adherens junctions, maintain the barrier function of endothelial cells (Claesson-Welsh et al. 2021; Cong and Kong 2020; Dejana et al. 2008; Higashi et al. 2020; Li et al. 2016; Nitta et al. 2003). Claudin-5 expression in aortic endothelial cells has been characterized by immunohistochemistry, in situ hybridization, and scRNA-seq analysis (Dekker et al. 2002; Engelbrecht et al. 2020). Claudin-5 expression is ubiquitous in brain vasculature but in most other organs is limited to arteries, arterioles, and some capillaries (Greene et al. 2019; Nitta et al. 2003; Richards et al. 2022). Our finding of little to no claudin-5 in the vena cava fits with the absence in venules but does not necessarily reflect the absence of tight junctions, as other junctional proteins can compensate (Nitta et al. 2003; Richards et al. 2022).

The mosaic pattern of claudin-5 is a distinctive feature of aortic endothelial cells that contrasts with the uniform distribution of VE-cadherin at endothelial cell borders (Engelbrecht et al. 2020; Kim et al. 2016; Richards et al. 2022). Although proof of the flow-dependency of the mosaic pattern of claudin-5 awaits evidence from studies similar to those used to define VE-PTP polarization (Mantilidewi et al. 2014; Shirakura et al. 2023), the correlated distributions of claudin-5 and VE-PTP polarization are consistent with a novel mechanistic link worth exploring between blood flow, VE-PTP, and endothelial barrier function.

## Conclusion

Endothelial cells of the thoracic aorta and vena cava are even more heterogeneous than previously recognized. Adding to the well documented specializations at branch sites, curvatures, and cardiac connections, endothelial cells of these vessels have a mosaic pattern of VE-PTP polarization in the downstream plasma membrane and cytoplasm. Tie2-pY992, a substrate of VE-PTP, has a similar mosaic pattern. Claudin-5 also has this pattern in the aorta but is nearly absent in the vena cava. Correlated mosaic patterns of VE-PTP polarization, Tie2-pY992, and claudin-5 in the aorta are consistent with coordinated effects of blood flow dynamics on endothelial barrier function. However, the presence of focal patches of leakage in the aorta compared to widespread leakage in the vena cava indicates additional barrier regulators. Together, the findings highlight the heterogeneous distributions of VE-PTP, Tie2-pY992, and claudin-5 in endothelial cells of the aorta and vena cava and the need for physiological measurements and intravital imaging to rigorously assess the contributions of blood flow dynamics and intrinsic differences in endothelial cells to the mosaic patterns.
